# Firing rate homeostasis counteracts changes in stability of recurrent neural networks caused by synapse loss in Alzheimer’s disease

**DOI:** 10.1371/journal.pcbi.1007790

**Published:** 2020-08-25

**Authors:** Claudia Bachmann, Tom Tetzlaff, Renato Duarte, Abigail Morrison

**Affiliations:** 1 Institute of Neuroscience and Medicine (INM-6) and Institute for Advanced Simulation (IAS-6) and JARA BRAIN Institute I, Jülich Research Centre, Jülich, Germany; 2 Institute of Cognitive Neuroscience, Faculty of Psychology, Ruhr-University Bochum, Bochum, Germany; University of Freiburg, GERMANY

## Abstract

The impairment of cognitive function in Alzheimer’s disease is clearly correlated to synapse loss. However, the mechanisms underlying this correlation are only poorly understood. Here, we investigate how the loss of excitatory synapses in sparsely connected random networks of spiking excitatory and inhibitory neurons alters their dynamical characteristics. Beyond the effects on the activity statistics, we find that the loss of excitatory synapses on excitatory neurons reduces the network’s sensitivity to small perturbations. This decrease in sensitivity can be considered as an indication of a reduction of computational capacity. A full recovery of the network’s dynamical characteristics and sensitivity can be achieved by firing rate homeostasis, here implemented by an up-scaling of the remaining excitatory-excitatory synapses. Mean-field analysis reveals that the stability of the linearised network dynamics is, in good approximation, uniquely determined by the firing rate, and thereby explains why firing rate homeostasis preserves not only the firing rate but also the network’s sensitivity to small perturbations.

## Author summary

Relating the properties of neuronal circuits with concrete functional roles pertaining to cognition and behavior is a complex endeavour. This is especially true when it comes to diseases and dysfunctions, where we are often left with high-level clinical observations (e.g. cognitive deficits and structural brain changes), without understanding their relationship. A potentially fruitful approach to address this problem consists of employing simplified mathematical models to test the relevant hypotheses, incorporating pathophysiological observations and evaluating their tentative functional consequences, in relation to clinical observations. In this work, we employ a spiking neural network model to study the effects of synaptic loss, as it is often observed in various neurodegenerative disorders, in particular Alzheimer’s disease (AD). We show that the loss of synapses drives the network into a less sensitive regime, which potentially accounts for the cognitive deficits of AD. We also endow the circuits with a compensation mechanism which restores the mean network activity by increasing the weight of the remaining connections. We demonstrate that this very simple compensatory mechanism can prevent the network from drifting into the less sensitive regime and recover all other dynamical features that are changed due to synapse loss. We further develop an analytical model that accounts for this surprising finding.

## Introduction

Accelerated synapse loss is a prominent feature in many types of neurodegenerative disorders, such as Huntington’s disease, frontotemporal dementia or Alzheimer’s disease [1–5]. In Alzheimer’s disease (AD), synapse loss appears to be particularly important, as it is widespread across different brain areas and constitutes a key marker in the AD pathology (see, e.g., [5]). The mechanisms underlying AD related synaptic modifications are currently the subject of intensive research, which has revealed that a number of different alterations at the molecular level may ultimately lead to synaptic decay [6–8], such as an abnormal occurrence of oligomeric and aggregated *β*-amyloid-peptides (A*β*), an abnormal phosphorylation of the tau protein and the occurence of neurofibrillary tangles, and a disrupted signaling in neuroinflammatory and oxidative stress responses [8–11].

Previous studies have uncovered a strong positive correlation between cognitive impairment in AD patients and synapse loss [12–19]. In contrast, correlations between the cognitive status and the density of plaques or tangles have frequently been reported as rather weak. Synapse loss is therefore not merely a structural epiphenomenon of AD, but appears to be *the* physical correlate of cognitive decline. While the most commonly reported early symptom of AD is memory deterioration, the disease is associated with a wide range of other cognitive problems such as stereotyped, repetitive linguistic production, visuo-spatial deficits and disorientation, apraxia, and loss of executive functions, i.e. planning and abstract reasoning [20, 21]. The observed progression of cognitive symptoms goes hand in hand with brain tissue atrophy [22–24] associated with loss of synapses [25], suggesting that the synaptic degeneration may underlie the cognitive deterioration following the gradual involvement of different, functionally specialized brain regions.

It is known that AD-related molecular and cellular alterations, such as abnormal depositions of A*β* plaques or atrophy rates, often significantly precede cognitive symptoms (see, e.g., [26, 27], and references therein). However, mechanisms exist that counteract synapse loss [28, 29], at least in the early stages of the disease. Various studies have shown that the loss of synapses is accompanied by a growth of remaining synapses, such that the total synaptic contact area (TSCA) per unit volume of brain tissue is approximately preserved [12, 17, 18, 30]. It is likely that such compensatory mechanisms underlie the observed delay in the onset of cognitive symptoms with respect to the onset of symptoms at the cellular level [31]. The heterogeneity in the disease progression and the propensity to transition from healthy cognitive aging to mild cognitive impairment and dementia may thus be associated to a subject’s ability to counteract synapse loss and, to a certain extent, maintain global functionality in a way that masks the progressive underlying pathophysiology. Such homeostatic, regulatory mechanisms appear to play an important role in counteracting structural deterioration and preserving computational capabilities. On the other hand, they pose important challenges to the network’s functionality since they have the potential to disrupt the specificities of a circuit’s microconnectivity (namely the distribution of synaptic strengths) and thus degrade its information content (e.g. [32]). Successful homeostatic compensation thus requires a balanced orchestration which preserves the system’s computational properties and macroscopic dynamics, e.g., average firing rates [33, 34] and E/I balance [35], as well as the relative ratios and distributions of synaptic strengths (e.g. synaptic scaling mechanisms; [36, 37]).

Understanding the circuit-level consequences of synaptic alterations, entailing both the deregulation by synapse loss and recovery through homeostasis, is essential to understand whether they represent a negative symptom of the disease or a compensatory response. One likely effect is the modification of the network’s firing rate. In order to maintain a physiological operating regime far from activity extremes (quiescence or epileptic activity), a network needs the capacity to regulate its firing rate. The degree to which this may be impaired in AD is still under debate (see [10, 38]). While the effects of synaptic alterations on the network dynamics have been partially characterized, a direct link between synapse loss, network dynamics and functional decline has yet to be systematically established, with only a few studies addressing the topic [39–41]. However, this connection may prove fruitful, both for understanding the disease itself and for fostering the development of new diagnostic and therapeutic approaches. It is currently unknown to what extent homeostatic mechanisms, such as increasing the synaptic area [12, 18], can completely recover the neuronal network’s firing rate, nor whether the preservation of the firing rate by such mechanisms entails the preservation of cognitive performance.

In this study, we investigate the link between structure, dynamics and function using a recurrent spiking neural network model [42]. Despite their simplicity, such systems have been shown to support computations, such as e.g. stimulus categorization, associative learning and memory, information routing and propagation, etc. (see e.g., [43–48]). Additionally, although these models have complex behavioral repertoires, they are often simple enough that their dynamics can be assessed analytically. The stability of the dynamics can then be related to computational task performance, such as the network’s sensitivity to perturbation and classification capability [49, 50]. Thus, an analytical treatment of network dynamics can provide insight into why some realizations of such networks perform better than others and how performance is affected by structural changes. Theoretical studies explicitly addressing this issue have so far focused either on the disruption of oscillations or functional connectivity of the whole brain, or on memory only (especially memory retrieval; [39–41]).

Here, we investigate how the loss of excitatory-excitatory synapses in sparsely connected random networks of spiking excitatory and inhibitory neurons (Sec. *Computational network model of Alzheimer’s disease*) and firing rate homeostasis, based on upscaling the remaining excitatory-excitatory connections, alters the dynamical characteristics of a network. Surprisingly, we find that firing rate homeostasis can restore a variety of dynamic features caused by synaptic loss, including the increase in spike train regularity, the drop in the fluctuations of population activity and the reduction of the synaptic contact area (Sec. *Total synaptic contact area and firing statistics*) caused by synaptic loss. In addition, we observe that synaptic loss decreases the network’s sensitivity to small perturbations (Sec. *Perturbation sensitivity and linear stability*), such that a network operating near the ‘edge of chaos’ would be shifted by synaptic loss to a more stable regime; a shift which has been shown in previous studies to result in a decrease in computational capacity [49–54], and may account for the cognitive deficits observed in Alzheimer’s disease. Here, too, firing rate homeostasis counteracts the shift towards the stable regime. We further show that these compensatory mechanisms ultimately become exhausted if physiological limits are placed on the growth of the synapse. As it is not obvious why simply maintaining the firing rate also maintains the stability of the network, we analyze the stability of the linearized network dynamics and discover a strictly monotonic relationship between the firing rate and the spectral radius of the network, which explains the restoration of the dynamics under the influence of firing rate homeostasis (Sec. *Perturbation sensitivity and linear stability*).

## Results

### Computational network model of Alzheimer’s disease

**Fig 1:**
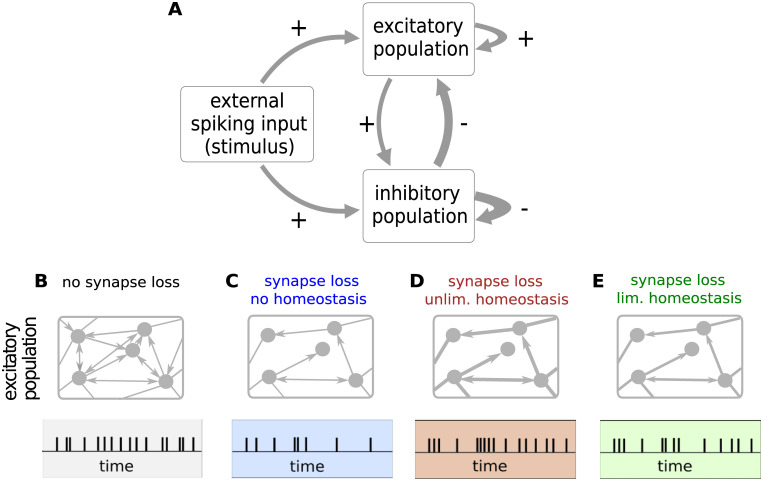
Sketch of the network model of Alzheimer’s disease and homeostasis. **A)** The network comprises two reciprocally and recurrently connected populations of excitatory (E) and inhibitory (I) integrate-and-fire neurons, excited by an external spiking input. Thickness of arrow indicates relative strength of the connection. In this study, Alzheimer’s disease is modeled by removing connections between excitatory neurons (loss of EE synapses) and upscaling of the remaining EE synapses to maintain the average firing rate (firing rate homeostasis). **B**–**E)** Sketch of EE connection density (number of arrows in upper panels), connection strength (thickness of arrows in upper panels) and resulting single-neuron spiking activity (lower panels). **B)** Intact network (without synapse loss). **C)** Synapse loss without homeostasis: removal of EE synapses and resulting reduction in firing rate. **D)** Synapse loss with unlimited homeostasis: removal of EE synapses and increase in strength of remaining EE synapses to maintain the average firing rate. Synaptic weights are allowed to grow without bounds. **E)** Synapse loss with limited homeostasis: removal of EE synapses and bounded increase in strength of remaining EE synapses. Here, synaptic weights cannot exceed 120% of their reference weight. The firing rate is therefore only partially recovered. For a complete description and parameter specification of the network model, see, Sec. *Network model* and, S1 and S2 Tables in the Supplementary Material.

We study the effects of AD related synaptic alterations on the network dynamics and computational characteristics in the framework of a generic mathematical neuronal network model (Fig 1A), which captures prominent structural and dynamical features of local neocortical networks such as the relative numbers of excitatory and inhibitory neurons [55, 56] and synapses [57, 58], sparse connectivity [56, 59], small synaptic weights [60], irregular [61–63] and predominantly asynchronous spiking [64], large membrane potential fluctuations [65–68], and a tight dynamical balance between excitatory and inhibitory synaptic currents [69]. The network is composed of randomly and sparsely connected populations of excitatory (E) and inhibitory (I) integrate-and-fire neurons, driven by external spiking input. The overall coupling strength is determined by the reference synaptic weight *J*. For simplicity, all excitatory connections (EE and IE) and all inhibitory connections (EI and II), respectively, have equal synaptic weight: *J*_EE_ = *J*_IE_ = *J* and *J*_EI_ = *J*_II_ = −*gJ* in the intact network (i.e. before synapse loss). The relative strength *g* of inhibitory weights is chosen such that the network is dominated by inhibition, to permit asynchronous irregular firing at low rates [70]. A complete specification of the network model and parameters can be found in Sec. *Network model* and S1 and S2 Tables in the Supplementary Material. An illustration of the connectivity of the excitatory population for an intact network and an example spike train is given in Fig 1B.

We implement the effects of AD on the network connectivity by reducing the number of excitatory synapses on excitatory neurons (EE synapses; [7, 71]), whilst keeping the number of connections between other populations (EI, IE, II) constant. In the absence of any compensation mechanism, this modification leads to a reduction in the average firing rate (see, Sec. *Total synaptic contact area and firing statistics*).

In biological neuronal networks, long-term activity levels are often stabilized by homeostatic regulation [20, 72, 73]. While a maintenance of firing rates has been observed at the level of individual neurons [33], long-term recordings suggest a predominance of a network-wide regulation [34] targeting a constant population firing rate. Such a homeostatic stabilization of the population firing rate can be accounted for by a global adjustment of synaptic weights (synaptic scaling; [74, 75]). Indeed, in the early stages of AD, synapse loss seems to be compensated by a growth of the remaining synapses [12, 18, 30]. To realize this mechanism in our spiking neuronal network, we implement a firing-rate homeostasis which compensates for the loss of EE synapses by a global increase in the weights *J*_EE_ of the remaining EE synapses, thereby preserving the population firing rate. (In order to demonstrate that our results also apply to other forms of homeostasis, we also implement a local synaptic scaling mechanism, in which the firing rate is regulated at the level of individual neurons.).

For advanced AD, where a large portion of the EE synapses has been lost, a full recovery of the population firing rate through synaptic scaling would require unrealistically large synaptic weights. During aging and dementia, the maximum increase in synaptic size has been reported to be in the range from 9% to 24% (see [17], and references therein). We incorporate these findings by introducing an optional upper bound for the weight *J*_EE_ of EE synapses.

To uncover the differential effects of excitatory synapse loss and homeostasis, in this study we investigate the dynamical and computational characteristics of a network for three different scenarios: synapse loss without homeostatic compensation (Fig 1C), synapse loss with an unlimited firing rate homeostasis where synaptic weights can grow without bounds (Fig 1D), and synapse loss with limited firing rate homeostasis where the synaptic weights cannot exceed 120% of the weight in the intact reference network (Fig 1E).

Note that the model’s high level of abstraction enables us to identify fundamental mechanisms, to reduce the risk of overfitting, and to arrive at general conclusions that may be transferred to other brain regions or even different spatial scales. Empirically observed features of biological neural networks such as heavy-tail synaptic weight distributions [76, 77] or active dendritic processing [78] are not explicitly incorporated. As a consequence, model parameters such as synaptic weights have to be regarded as “effective” parameters and cannot be mapped to biological parameters in a one-to-one fashion. Selecting a particular set of parameters to be considered “biologically realistic” would be misleading. Therefore, rather than focusing on a specific configuration of the model, we systematically vary both the reference synaptic weight *J* and the extent of synapse loss to uncover the general relationship between these parameters and the dynamical and computational properties of the network.

**Fig 2:**
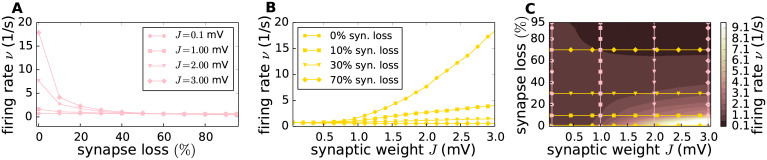
Effect of different parameter configurations on the network’s firing rate. The time and population-averaged firing rate is explored with respect to two parameters: the synaptic weight *J* and the loss of EE synapses. In **A** the EPSP amplitude is fixed (*J* ∈ {0.1, 1, 2, 3}mV, represented by a circle, square, triangle and diamond markers, respectively) and the firing rate *ν* is plotted against various degrees of synapse loss. In **B** the firing rate *ν* is plotted against the synaptic weight *J* for four different degrees of EE synapse loss (0%, 10%, 30%, 70%, with circle, square, triangle and diamond markers). The information of these two plot is combined in the contour plot **C**, which shows the dependence of the firing rate *ν* (colour coded) on synaptic reference weight *J* and the degree of synapse loss. Vertical rose lines correspond to the lines plotted in **A**, horizontal lines to the yellow lines in **B**. For visualization purposes, markers show only a subset of the data. All plotted data corresponds to the mean across 10 random realizations.

Fig 2 demonstrates the main effects of varying these parameters. The firing rate of a network increases monotonically with the choice of reference synaptic weight *J*; for a network with a given *J*, the firing rate of the network decreases with the loss of EE synapses, with the majority of the rate reduction occurring in the range 0–20% (Fig 2A). Likewise, for a given degree of synaptic loss, the firing rate increases with the strength of the remaining EE synapses (Fig 2B). Throughout this work, we investigate the behaviour of the network across the two demensional parameter space spanned by the reference synaptic weight *J* and the extent of synapse loss. The results are typically visualized with the help of contour plots whose interpretation in the context of variations in firing rate is illustrated in Fig 2C, in order to facilitate their comprehension. Note that a reduction of synapses of 95% is of course not biologically plausible; we chose generous ranges for both parameters to give a better visulization of the behaviour of the system and to demonstrate the surprisingly strong effects of homeostasis.

### Total synaptic contact area and firing statistics

**Fig 3:**
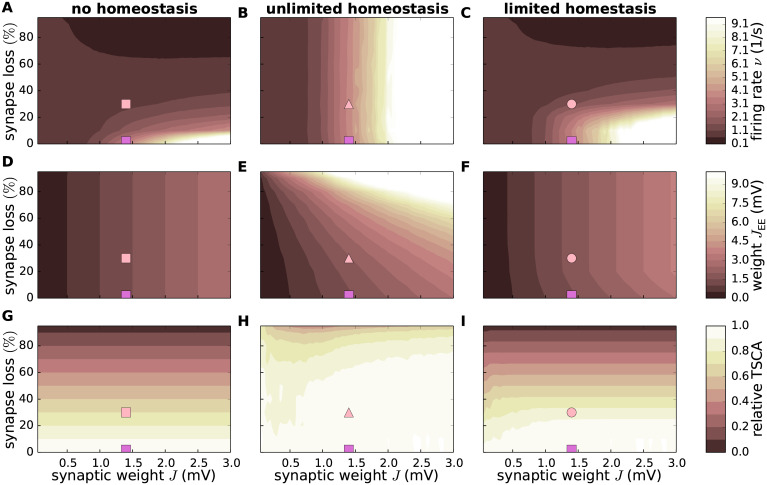
Effect of synapse loss and firing rate homeostasis on firing rate, synaptic weights and total synaptic contact area. Dependence of the time and population averaged firing rate *ν* (**A**–**C**), synaptic weight *J*_EE_ (**D**–**F**) and the relative total synaptic contact area (TSCA) of EE synapses (**G**–**I**) on the reference weight *J* and the degree of EE synapse loss in the absence of homeostatic compensation (left column), as well as with unlimited (middle column) and limited firing rate homeostasis (right column). Color-coded data represent mean across 10 random network realizations. Symbols mark parameter configurations shown in Fig 5.

In the absence of homeostatic compensation (left column of Fig 3), removal of excitatory synapses on excitatory neurons naturally results in a decrease in the population firing rate *ν*, irrespective of the synaptic-weight scale *J* (Figs 2 and 3A). An upscaling of the remaining EE synapses (middle column) allows us to preserve the population firing rate, even if substantial amounts of synapses are removed (vertical contours in Fig 3B). If the maximum synaptic weight is limited, firing rates are preserved only up to a critical level of synapse loss (early stages of AD; Fig 3C).

Experimental studies have shown that, in early AD, the reduction in the number of synapses is accompanied by a growth of the remaining synapses such that the total synaptic contact area (TSCA) per unit volume is approximately preserved [12, 18, 30]. Our simple AD network model reproduces this finding if we define the TSCA as the product of the number of EE connections and the synaptic weight *J*_EE_ (Sec. *Synaptic contact area and characterization of network activity*). Without homeostatic upscaling of EE weights, the TSCA is proportional to the number of EE connections and therefore quickly decreases with increasing levels of synapse loss (Fig 3G). In the presence of firing rate homeostasis, however, the TSCA remains largely constant unless a majority of synapses is lost (Fig 3H) or the maximum synaptic weight is reached (Fig 3I). We conclude that the experimentally observed stabilization of the TSCA in the face of synapse loss may be a consequence of a homeostatic synaptic scaling regulated by the average population firing rate.

**Fig 4:**
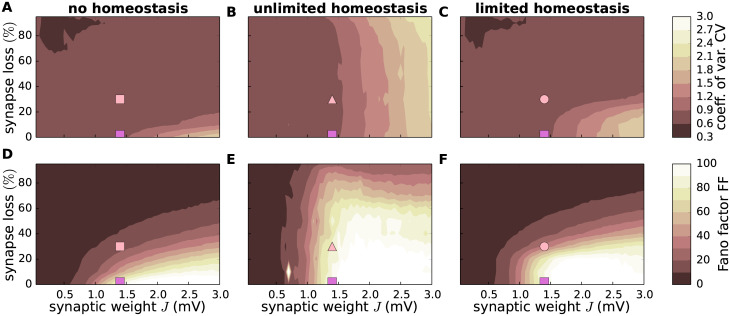
Effect of synapse loss and firing rate homeostasis on spike train statistics. Dependence of the coefficient of variation CV of inter-spike intervals (**A**–**C**) and the Fano factor FF of the population spike count (binsize *b* = 10ms; **D**–**F**) on the synaptic reference weight *J* and the degree of EE synapse loss in the absence of homeostatic compensation (left column), as well as with unlimited (middle column) and limited firing rate homeostasis (right column). Color-coded data represent mean across 10 random network realizations. Symbols mark parameter configurations shown in Fig 5.

In physiologically relevant low activity regimes, neuronal firing is determined both by the mean as well as by fluctuations in the synaptic input. A reduction in the number of synapses followed by an upscaling of synaptic weights may preserve the average population firing rate; it cannot, however, simultaneously preserve the mean and the variance of the synaptic input currents. The neurons’ working point, i.e. the statistics of the synaptic input, will inevitably change. A priori, it is therefore not clear to what extent synapse loss and firing rate homeostasis alter the overall firing statistics in the recurrent network beyond the average firing rate. Here, we address this question by studying the irregularity of spike generation by individual neurons, measured by the coefficient of variation CV of the inter-spike interval distribution, and spike-train synchrony, assessed by the normalized variance of the population spike count, the Fano factor FF, in 10ms time intervals (see, Sec. *Synaptic contact area and characterization of network activity*). Without homeostatic compensation, synapse loss generally results in spike patterns that are less irregular (Fig 4A) and less synchronous (Fig 4D). In the presence of firing rate homeostasis, however, both the CV and the FF are largely preserved (Fig 4B and 4E; light spot in E is due to a significant outlier in one simulation). Only if the level of synapse loss becomes too severe or if the synaptic-strength limits are reached (limited homeostasis), the CV and the FF are reduced (Fig 4B, 4C, 4E and 4F).

**Fig 5:**
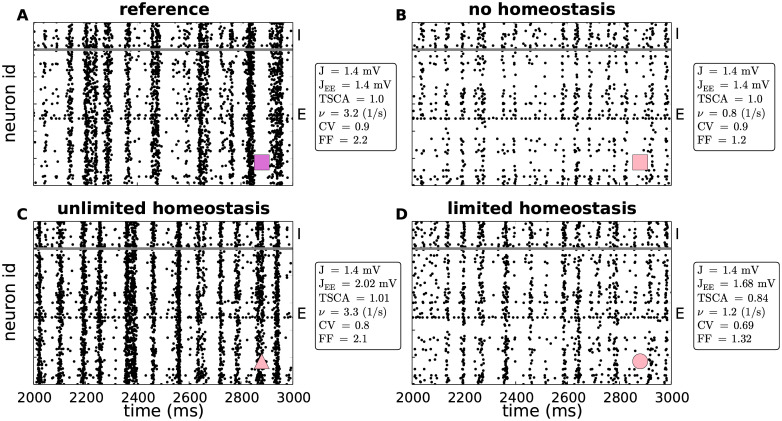
Effect of synapse loss and firing rate homeostasis on spiking activity. Spiking activity (dots mark time and sender of each spike) in an intact reference network (no synapse loss, *J*_EE_ = 1.4mV; **A**), as well as in networks where 30% of the EE synapses are removed: **B)** no homeostasis (*J*_EE_ = 1.4mV), **C)** unlimited homeostasis (*J*_EE_ = 2.02mV), **D)** limited homeostasis (*J*_EE_ = 1.68mV). In all panels, the synaptic-weight scale is set to *J* = 1.4mV. Examples depict parameter configurations marked by corresponding symbols in Figs 3 and 4 (cf. marker in lower right corner of each panel). The values for the parameters explored in these figures (synaptic weight *J*, EE synaptic weight *J*_EE_, total synaptic contact area *TSCA*, firing rate *ν*, coefficient of variation CV and Fano factor FF) are listed next to each plot. Regions below and above the gray horizontal line show spiking activity of a subset of 100 excitatory (E) and 25 inhibitory neurons (I), respectively.

For illustration, Fig 5 depicts the spiking activity for four example parameter settings marked by the symbols in Figs 3 and 4. As Fig 4E and 4H already suggest, the overall spiking activity, e.g the number and the duration of synchronous event and the spiking frequency of single neurons, of the homeostatic network (Fig 5C) and the reference network (Fig 5A) are very similar. Only the exact timing of the synchronous events and the single neuron spiking differ. In the AD network without homeostasis (Fig 5B), the firing rates of both excitatory and inhibitory neurons are decreased. The number of synchronous events, compared with the reference network (Fig 5A), does not seem to be decreased, but their duration does. The network with limited homeostasis (Fig 5D) is more similar to the AD network without homeostasis than the unlimited homeostasis networks, because the restriction in synaptic growth prevents the rate from being recovered.

### Perturbation sensitivity and linear stability

An open question in Alzheimer’s disease research is how cellular damage such as synapse loss affects patients’ cognitive capabilities. A number of theoretical studies have shown that recurrent neuronal networks exhibit optimal computational performance characteristics for a variety of task modalities if they operate in a dynamical regime where small perturbations are neither instantly forgotten nor lead to entirely different network states [49–54]. In dynamical systems theory, this regime has been termed the “edge of chaos” as it represents the transition from a stable state with a low sensitivity to small perturbations to a chaotic state where the sensitivity to small perturbations is high. Here, we investigate the role of synapse loss and firing rate homeostasis for the network’s sensitivity to perturbations as an indicator of its overall computational performance.

**Fig 6:**
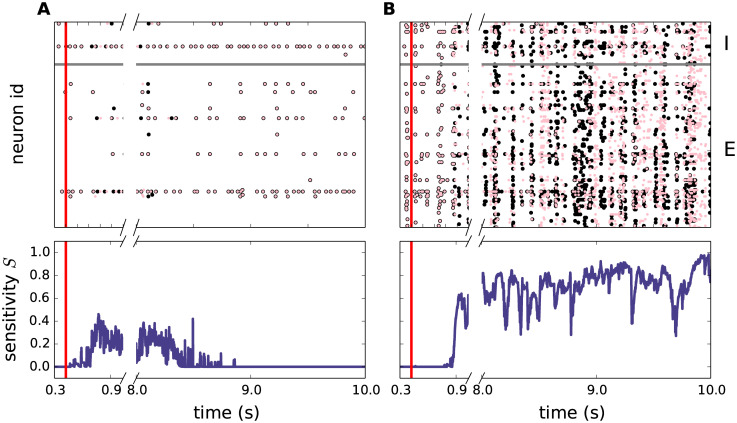
Perturbation sensitivity. Top: Example spiking activity (dots mark time and sender of each spike) of two identical networks (identical neuron parameters, connectivity, external input, initial conditions) with (black dots) and without perturbation (purple dots). The perturbation consists in delaying one external input spike at time t* = 400ms by δt* = 0.5ms. The vertical red line marks the time of the perturbation. Spikes of only 10% of all neurons are shown. Neurons below and above the horizontal gray line correspond to excitatory and inhibitory neurons, respectively. Bottom row: Perturbation sensitivity *S*(*t*) = 1 − |*R*(*t*)| obtained from the correlation coefficient *R*(*t*) of the low-pass filtered spike trains generated by the unperturbed and the perturbed network (black and purple dots in top panels; see, Sec. *Synaptic contact area and characterization of network activity*). **A)** Stable dynamics (*J* = 0.45mV, *K*_EE_ = 100). **B)** Chaotic dynamics (*J* = 1.75mV, *K*_EE_ = 100).

To assess the perturbation sensitivity, we simulate a given network twice with identical initial conditions and identical realizations of external inputs. In the second run, we apply a small perturbation by delaying one of the external input spikes to a single neuron by a fraction of a millisecond (Fig 6). In stable regimes, the effect of this perturbation on the spiking response is transient and quickly vanishes (Fig 6A, top). In chaotic regimes, in contrast, the small perturbation leads to diverging spike patterns (Fig 6B, top). We quantify the network’s perturbation sensitivity *S* = 1 − |*R*| in terms of the long-term correlation coefficient *R* between the low-pass filtered spike responses in the two runs (Fig 6, bottom). With this definition, *S* = 0 and *S* = 1 correspond to insensitive (stable) and highly sensitive (chaotic) networks, respectively (for details, see, Sec. *Synaptic contact area and characterization of network activity*).

**Fig 7:**
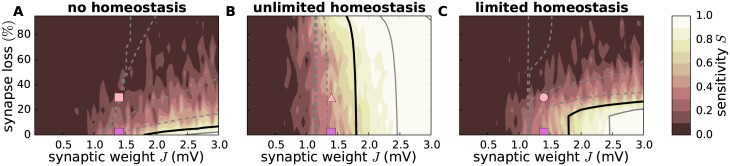
Effect of synapse loss and firing rate homeostasis on perturbation sensitivity. Dependence of perturbation sensitivity *S* on the synaptic reference weight *J* and the degree of EE synapse loss in the absence of homeostatic compensation (**A**), as well as for unlimited (**B**) and limited firing rate homeostasis (**C**). Color-coded data represent mean across 10 random network realizations. Superimposed black and gray curves mark regions where the linearized network dynamics is stable (gray dashed; spectral radius *ρ* = …, 0.6, 0.8), about to become unstable (black; *ρ* = 1), and unstable (gray solid; *ρ* = 1.2, 1.4, …). Pink symbols mark parameter configurations shown in Figs 3 and 5.

For small synaptic weights *J*, the network dynamics is always stable (*S* = 0) for our choice of parameters, irrespective of the degree of synapse loss and the absence or presence of homeostatic compensation (Fig 7). In this regime, the perturbation has no long-term effect: after a transient phase, the response spike patterns in the perturbed and the unperturbed simulation are exactly identical (at the temporal resolution Δ*t*_f_ = 1ms of the recorded signals). The intact networks (zero synapse loss) enter a chaotic regime (*S* > 0) if the synaptic weights *J* exceed a certain critical value. Removal of EE synapses without homeostatic compensation leads to a shift of this transition towards larger synaptic weights (Fig 7A). Networks in the chaotic regime eventually become insensitive to perturbations with progressing EE synapse loss. In the presence of firing rate homeostasis, in contrast, the perturbation sensitivity is preserved (color gradient in Fig 7B is predominantly left to right, rather than top to bottom). Unless the homeostatic strengthening of EE synapses is limited (limited homeostasis; Fig 7C and S3 Fig in the Supplementary Material), this maintenance of the perturbation sensitivity is observed even if the degree of synapse loss is substantial (> 80%).

We conclude that synapse loss, as observed in Alzheimer’s disease, tends to reduce the perturbation sensitivity of the affected networks, and may thereby impair their computational performance for a broad range of task modalities. Homeostatic mechanisms that preserve the average network activity (firing rate) can prevent this reduction in sensitivity and, hence, the decline in computational capability.

So far, the reported results on the perturbation sensitivity were obtained by network simulations for a specific set of parameters. In the following, we employ an analytical approach; firstly, to show that our findings are general and do not depend on the details of the network model, and secondly, to shed light on the mechanisms underlying the reduction in perturbation sensitivity by synapse loss and its maintenance by firing rate homeostasis.

As shown in [79], the dynamics of large random networks of analog nonlinear neurons without (or with constant) external input undergoes a transition from a stable to a chaotic regime at some critical synaptic coupling strength. The study further revealed that this transition coincides with a critical point where the local linearized network dynamics becomes unstable. For more realistic networks of spiking neurons, networks with fluctuating external input or networks with a more realistic connectivity structure, a strict correspondence between the onset of chaotic dynamics and linear instability could not be established [54, 80–84]. Nevertheless, various previous studies suggest that the two transition types are interrelated, in the sense that a change in the linear stability characteristics is accompanied by a change in the network’s sensitivity to small perturbations.

Here, we propose that the linear stability characteristics can serve as an indirect and easily accessible indicator of the network’s sensitivity to small perturbations, and hence its computational capability. As described in Sec. *Linearized network dynamics and stability analysis*, the linearized network dynamics is determined by the effective connectivity matrix ***W***. Its components *w*_*ij*_ = ***W***_*ij*_ (*i*, *j* ∈ {1, …, *N*}) measure the effect of a small fluctuation in the firing rate *ν*_*j*_(*t*) of a presynaptic neuron *j* on the rate *ν*_*i*_(*t*) of the postsynaptic neuron *i* at a specific working point determined by the stationary firing rates *ν* = (*ν*_1_, …, *ν*_*N*_). The effective connection weights are hence determined not only by the synaptic weights *J*_*ij*_, but also by the excitability of the target cell *i*, which is in turn determined by the statistics of the synaptic input fluctuations, i.e. the dynamical state of the local network. The linearized dynamics becomes unstable if the spectral radius *ρ* = Re(λ_max_), the real part of the maximal eigenvalue λ_max_ of ***W***, exceeds unity.

Loss of EE synapses corresponds to setting a fraction of the excitatory components *w*_*ij*_ (i,j∈E) to zero. In the absence of homeostatic compensation, we expect this weakening of positive feedback to have a stabilizing effect. The dependence of the effective weights *w*_*ij*_ on the working point, however, leads to a non-trivial effect of synapse loss and firing rate homeostasis on the spectral radius *ρ*. Here, we compute *ρ* by employing the diffusion approximation of the leaky integrate-and-fire neuron and random-matrix theory (for details, see, Sec. *Linearized network dynamics and stability analysis*).

As shown in Fig 7 (black and gray curves), the linear stability characteristics (as measured by the spectral radius *ρ*) bear striking similarities to the sensitivity to perturbations. In the absence of homeostasis, loss of EE synapses leads to a fast decrease in *ρ*. Linearly unstable networks quickly become stable (Fig 7A). Firing rate homeostasis, in contrast, preserves the spectral radius *ρ*, even if a substantial fraction of EE synapses is removed. Linearly unstable networks remain unstable (Fig 7B), until the homeostatic resources are exhausted (Fig 7C).

**Fig 8:**
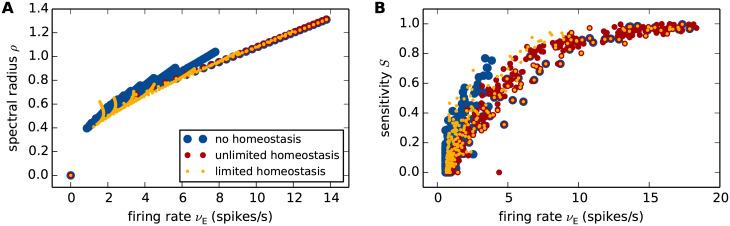
Firing rate as predictor of linear stability and perturbation sensitivity. Dependence of the linear stability quantified by the spectral radius *ρ* (**A**; theory) and the perturbation sensitivity *S* (**B**; simulation results) on the mean stationary firing rate *ν*_E_ of the excitatory neuron population in the absence of homeostasis (blue), as well as for unlimited (red) and limited firing rate homeostasis (yellow). Scatter plots depict data for various reference weights *J* ∈ {0, …, 3}mV and various degrees of synapse loss from 0% to 50%. Same data as in Figs 7, 12D–12F and 12M–12O.

The analytical approach described in Sec. *Linearized network dynamics and stability analysis* provides us with an intuitive understanding of why and under what conditions firing rate homeostasis preserves the linear stability characteristics in the face of synapse loss. The analysis shows that, in the presence of firing-rate homeostasis, the spectral radius *ρ* is uniquely determined by the stationary average firing rate (red points in Fig 8A and Eq 18). For the parameters chosen in this study, an approximately unique dependence on the firing rate is also observed in the absence of homeostasis and for limited homeostasis (blue and yellow points in Fig 8A). Network simulations reveal similar findings for the perturbation sensitivity *S* (Fig 8B). For unlimited homeostasis, the firing rate, the perturbation sensitivity and the spectral radius remain (approximately) constant during synapse loss (red points in Fig 8). In the absence of homeostasis or for limited homeostasis, firing rates change; however, the relationship between firing rate and spectral radii *ρ* and perturbation sensitivities *S* nevertheless remains constant (red, blue and yellow points in Fig 8 lie over one another). The number *K*_EE_ and the strength *J*_EE_ of EE synapses therefore play only an indirect role by determining the stationary firing rate *ν*. Any combination of *K*_EE_ and *J*_EE_ that preserves *ν* will simultaneously preserve *ρ* (and *S*).

The unique dependence of the spectral radius *ρ* on the firing rate *ν* is a consequence of the working-point dependence of the effective weights *w*_*ij*_ ≈ *η*(*ν*_*i*_)*J*_*ij*_/*σ*_*i*_, where *η*(*ν*_*i*_) is a function of the firing rate *ν*_*i*_ of the target neuron *i* (see Eq 16). To maintain the stationary firing rate *ν*_E_ of excitatory neurons, the synaptic weights *J*_EE_ are increased to compensate for the loss of excitatory synapses, i.e. for the decrease in the number *K*_EE_ of excitatory inputs. This increase in the synaptic weights *J*_*ij*_ (for neurons *i*, *j* both in the excitatory population) is accompanied by an increase in the variance $\sigma_{i}^{2}$ of the synaptic input received by the target neuron *i*. If the response firing rate *ν*_*i*_ is kept constant (as is the case in the presence of firing rate homeostasis), an increase in *σ*_*i*_ leads to a decrease in neuron *i*’s sensitivity to a modulation of the input current caused by a spike of the source neuron *j*. This interplay between an upscaling of the weights *J*_*ij*_ and a downscaling of the neuron’s modulation sensitivity restricts the growth in the effective weight *w*_*ij*_, and, ultimately, leads to a preservation of the spectral radius *ρ*. In Sec. *Linearized network dynamics and stability analysis*, we demonstrate this effect for a homogeneous network of leaky-integrate-and-fire neurons. The derivation relies on the assumption that the synaptic weights are sufficiently small and the rate of synaptic events is high (diffusion approximation), that the stationary firing rates *ν*_E_ and *ν*_I_ of excitatory and inhibitory neurons are identical (homogeneity), and that the input fluctuations caused by external sources are small compared to those generated by the local network.

### The interplay of hypo- and hyperactivity and its effect on E/I balance and perturbation sensitivity

Despite the broad scope of pathological changes observed in AD, we have only considered the effect of synapse loss in our AD model so far. The reason for this simplification is the strikingly high correlation between synapse loss and cognitive decline in AD [12–19], which suggests it plays a particularly prominent role in AD’s pathophysiology. We have shown that the loss of EE synapses leads to a decreased firing rate (hypoactivity, see, Sec. *Total synaptic contact area and firing statistics*). Our theoretical analysis shows that this effect also generalises to the unspecific loss of synapses (see, S2 Fig in Supplementary Material). Another, seemingly contradictory observation, is the occurrence of hyperactive episodes (increased network activity, epileptic discharges) that predominantly take place at the initial stages of the disease [85–91]. It has been argued that this hyperactivity is one of the main disease triggers, being responsible for a broad range of subsequent pathologic alterations, such as changes in synaptic receptor expression, synapse loss and neuronal degeneration (for review, see [92]). Accordingly, hyperactivity, which coincides with a shift of the E/I balance towards excitation [93], has been studied intensively and several potential mechanistic causes have been suggested. For example, amyloid beta has been shown to increase glutamate release [94]. Also the loss of particular connection, e.g. from excitatory to inhibitory neurons [95] or from inhibitory to excitatory neurons [90, 96], might contribute to an increased overall network connectivity.

The manifold and sometimes contradictory empirical observations make it impossible to create a computational AD model that is in conformity with all findings. Therefore, we choose to focus on the most established and uncontroversial phenomena. First, hyperactivity is followed by hypoactivity with some hyperactive neurons/episodes emerging at later disease stages [85, 86, 92]. Second, increased synaptic volume [12, 17, 18, 30] correlates with increased postsynaptic potentials and characterize the initial stages of the disease [30, 92]. Third, synapse loss has been observed in all disease stages and appears to be the best correlate of cognitive decline [12–19, 97].

These well-documented observations, could be accounted for by two possible scenarios. In the first scenario, AD-specific changes in the brain primarily lead to synapse loss and hypoactivity, which is compensated for by increased synapse growth and other compensatory mechanisms. These homeostatic mechanisms might be insufficiently regulated, resulting in episodes of hyperactivity. As the disease progresses, the resources that compensate for synapse loss become exhausted and hypoactivity prevails. In the second potential scenario, AD triggers alterations that cause hyperactivity such as the growth of EE synapses or the weakening of particular connections that promote inhibition (presumably synapses from inhibitory to excitatory neurons). A compensatory reaction of the system then reduces the number of EE synapses in order to increase the impact of inhibition (or decrease the total excitation). This compensation may overshoot, resulting in a pathologic hypoactivity in the later stages of the disease.

**Fig 9:**
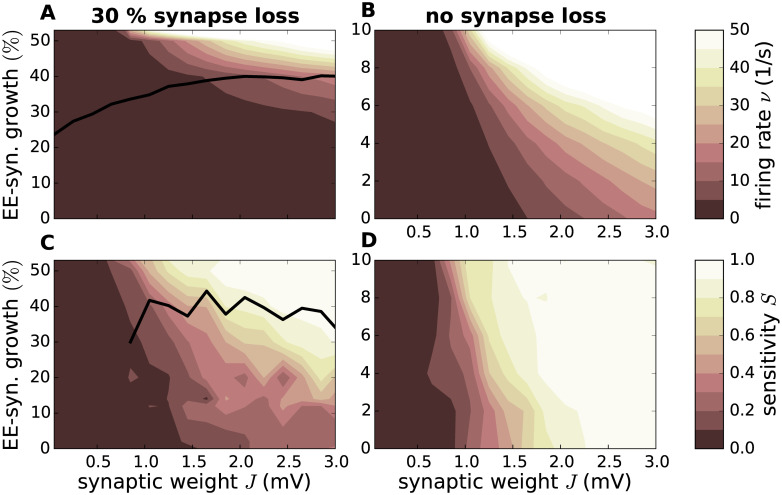
Effect of EE synapse growth on firing rate and sensitivity. Dependence of the firing rate **(A, B)** and the sensitivity **(C, D)** on the synaptic reference weight and the degree of EE synapse growth for an EE synapse loss of 30% (*K*_EE_ = 70) **(A, C)** and no synapse loss **(B, D)**. An EE synapse growth of zero means that all excitatory synapses have the same weight (*J*_EE_ = *J*_IE_). For values larger than zero, synapses of excitatory neurons onto other excitatory neurons are stronger than onto inhibitory neurons. Black curve in **A** and **C** represent the firing rate and sensitivity of the reference network (no synapse loss, *J*_EE_ = *J*_IE_). Color-coded data represents mean across 10 random network realizations.

So far, we have only considered the first scenario (EE synapse loss and EE synapse growth as a compensation mechanism) without modeling an overshooting compensation that leads to hyperactivity. Here, we examine the consequences of such a deregulated homeostasis and excessive increment of the EE synaptic weights in an AD network that already lost EE connections (*K*_EE_ = 70). As described in Sec. *Network model*, this increase is not, as previously the case, limited by a reference firing rate. As Fig 9A shows, increasing the EE weights beyond the point in which the network reaches its reference firing rate (black line), easily leads to an explosion of the firing rates. This rise in firing rate goes hand-in-hand with an increased sensitivity (Fig 9C), which likewise exceeds the sensitivity of the reference network at similar points (black line).

As a comparison, we examine the effects of hyperactivity according to the second scenario, i.e. as the primary disease trigger and not as a consequence of an homeostasis overreaction, by increasing the weight of EE synapses in a fully connected network (*K*_EE_ = 100). Our results show that even a comparably small increase to the strength of the EE synapses can increase the network’s firing rate drastically, once again causing the network to become more sensitive to small perturbations (Fig 9B and 9D).

A possible homeostatic response to such an increased activity is a reduction in the number of EE synapses. To give numeric examples, in a network with a reference synaptic weight of *J* = 0.85mV subject to synapse loss of 30% which is overcompensated for by increasing the EE synapse weight by 50%, the firing rate raises form 1.2 spikes/s to about 2.6 spikes/s. Simultaneously, the sensitivity increases from 0.06 to 0.16 (data extracted from Fig 9A and 9C). A homeostatic reduction of EE synapses to regain the original firing rate of 1.2 spikes/s results in a network with a net loss of 38%, but with the reference sensitivity of 0.06.

If the same reference network (*J* = 0.85mV) increases its EE weight by 10% the firing rate raises from 1.2 spikes/s to 7.7 spikes/s accompanied by a shift in the sensitivity from 0.06 to 0.43 (data extracted from Fig 9B and 9D). If the network compensates for this with an 11% loss of synapses, both the firing rate and the sensitivity of the reference network are recovered. These examples demonstrates that the order of hyper/hypoactivity, synapse loss and growth is not important for the sensitivity of the network. The only relevant quantity is the firing rate to which the network converges.

**Fig 10:**
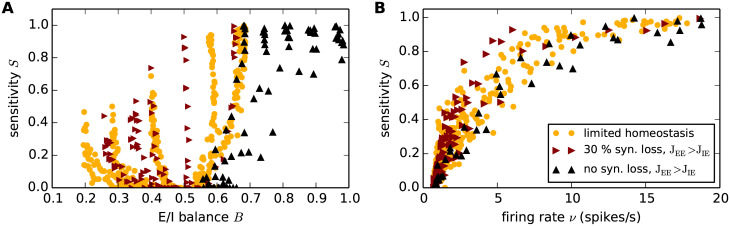
Firing rate and not E/I balance as predictor of linear stability and perturbation sensitivity. Dependence of perturbations sensitivity *S* on the E/I balance *B* **(A)** and on the mean stationary firing rate **(B)** for the limited homeostasis network (compare Fig 8, yellow), a network of full synaptic density (*K*_EE_ = 100) but increased EE synapse growth (*J*_EE_ ∈ {1 ⋅ *J*_IE_, …, 1.1 ⋅ *J*_IE_}; black) and for a network in which 30% EE synapse loss (*K*_EE_ = 70) is compensated by EE synapse growth (*J*_EE_ ∈ {1 ⋅ *J*_IE_, …, 1.5 ⋅ *J*_IE_}; red). Color-coded data represents mean across 10 random network realizations.).

In addition to the question of the mechanisms causing hyperactivity in AD, much consideration has been given to its effects. Hyperactivity has been put in the context of a disruption of the network’s E/I balance [93], which is assumed to make a major contribution to cognitive decline [98, 99]. This implies that an alteration in the E/I balance automatically implies a change in computational performance. To investigate this hypothesis, we examine the relationship of the sensitivity of the network to the E/I balance of the reference and hyperactive networks (estimated as described in Sec. *Synaptic contact area and characterization of network activity*). Fig 10A shows that networks exhibit a large range of sensitivities for a given E/I balance, in comparison to a rather narrow range for a given firing rate (Fig 10B). We thus conclude that firing rate is the primary indicator of the sensitivity of a network, rather than E/I balance.

### Effect of local homeostasis on perturbation sensitivity

The homeostatic regulation of the firing rate seems to take place on different spatial scales in the mammalian cortex: within each neuron or even within each dendrite [33, 100], and across a group of neurons in a network [34]. So far, we have only considered a global (network-wide) homeostatic regulation of the network’s firing rate in which the adjustment of a single model parameter (the EE weight) was sufficient to achieve the reference state. For the investigations described above, the necessary alteration to the parameter was determined in an offline fashion according to the following procedure: simulation of the reference network to establish the reference firing rate; deletion of synapses to create an AD network; increase of strength of remaining synapses of the AD network until the reference rate was reached; simulation of the AD network with the new weight configuration. Naturally, this scenario does not reflect what happens in the brain, in which homeostatic regulation occurs continuously.

In this section, we examine to what extent our main result (the sensitivity to perturbation as a unique function of the firing rate) is robust with respect to a local mechanism of firing rate homeostasis combined with a continuous weight update, as proposed by [101, 102]. As described in Sec. *Network model*, we first delete EE synapses and then simulate the network equipped with the local homeostatic mechanism for an additional 1200sec. During that time, each excitatory neuron attempts to reach its target firing rate by increasing incoming excitatory synapses if its rate is below the target rate, and deleting them if its rate is above the target rate. New synapses are created either with the same weights as the existing synapses in the reference network or, assuming a process of synaptic growth, with a very small weight. Shortly before the end of the simulation, we measure the firing rates of the neurons and sensitivity of the network as described in Sec. *Network model*.

**Fig 11:**
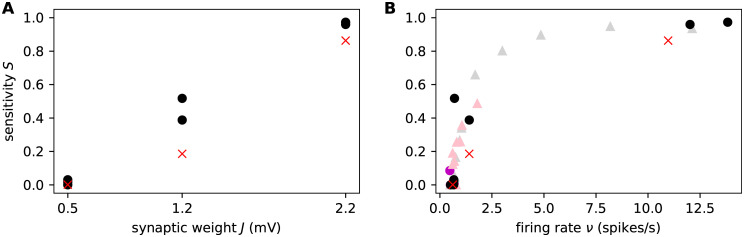
The effect of local homeostasis on sensitivity. Dependence of sensitivity to perturbations of networks with local homeostasis on different synaptic reference weight *J* ∈ {0.5mV, 1.2mV, 2.2mV} **(A)** and on the network’s population average firing rate *ν*. Red crosses: reference networks without synapse loss (*K*_EE_ = 100); black dots: networks that underwent synapse loss but that regained the reference firing rate, assuming new synapses were created with a weight of *J*_lh_ = *J*_EE_; pink dots: as for black dots, but initial synapses created with a weight of *J*_lh_ = 6 ⋅ 10^−4^mV. **(B)** relationship between sensitivity and firing rate for all networks, regardless of whether the original firing rate was regained. Dots and circles as in **(A)**; gray and pink triangles: as for black and pink dots, but for networks that could not restore the reference rate. All data points represent the mean across all realizations of a particular network weight *J* and a particular degree of synapse loss (0, 20, 40, 60, 80%).

Fig 11A shows that if the local homeostasis model succeeds in regaining the rate of the corresponding reference network, then the sensitivity of the reference network is also regained. Conversely, those networks that do not converge to the desired activity state do not exhibit the sensitivity of the reference network. Unsurprisingly, networks in which new synapses are very small are less likely to converge to the reference rate in the time allowed. Irrespective of whether the target rate is reached, the sensitivity of the network is determined by its firing rate, as demonstrated in Fig 11B. We thus conclude that our main finding is robust to the assumption of global or local homeostasis mechanisms.

## Discussion

In this article, we study the effect of Alzheimer’s disease on the dynamics and perturbation sensitivity of recurrent neuronal networks. To this end, we employ a computational model of a generic neuronal network composed of excitatory and inhibitory spiking neurons. Alzheimer’s disease is implemented, to a first approximation, in the form of a loss of excitatory synapses onto excitatory neurons. The resulting decrease in the firing rate is avoided (or delayed) by firing rate homeostasis, which is achieved by increasing the weights of the remaining excitatory-excitatory (EE) synapses. In one scenario, we allow synaptic weights to grow without bounds; in another, to ensure that they stay within the physiological range [18], we limit the maximum synaptic weight during homeostasis to 120% of the reference weight in the intact network (i.e. before synapse loss). We show that, in the absence of homeostatic compensation, a progressive loss of EE synapses not only reduces the average firing rate, but also leads to an increase in spike train regularity and a decrease in the fluctuations of the population activity.

This reduction in firing rate appears to be at odds with empirical observations, which demonstrate that network activity is enhanced in the areas affected earlier (e.g. hippocampus) [87, 103, 104]. For the observations of hyperactive states dominating the initial stages of the diseases and hypoactive brain activity combined with excessive synapse loss in the later stages, two possible scenarios are discussed in literature [18, 92]. In the first scenario, hyperactivity occurs in the first place and is compensated by activity-reducing strategies, e.g. synapse loss. These compensation mechanisms overshoot leading to hypoactivity in later disease stages. The physiological observation supporting this hypothesis is that oligomeric Amyloid-beta (A*β*) aggregates primarily boost glutamate release and change its uptake [92]. On the long run, glutamate spillover and the potential of A*β* oligomers to enhance the occurrence of phosphorylated tau in spines (for review, see [8]), causes degradation of synaptic connections [105]. This process may justify the decreased activity, as predicted by our model, during later stages of the disease, when the tau-pathology becomes more prominent (see, e.g., [86, 91, 106, 107]). In the second scenario, hypoactivity primarily caused by synapse loss is compensated by changes that increase activity. Dysregulated compensatory mechanisms would then lead to hyperactive states. In the course of the disease, compensatory mechanisms are stretched to their limits and cannot compensate for the excessive synapse loss anymore. Both scenarios have in common that: a) if homeostatic mechanism are optimal, such that the physiological firing rate is maintained, the resulting network configurations feature stronger but fewer EE synapses; b) synapse loss and hyperactivity dominates at later disease stages.

Because of these two commonalities and the possibility to draw analogies between the two different models, we focus our analysis only on the second scenario, in which synapse loss happens first. According to our AD model, the decrease in firing rate in more advanced disease stages can be delayed by homeostatic synaptic scaling. Moreover, our model predicts that, as long as the homeostatic mechanisms are able to restore the network’s firing rate, the CV and Fano factor are also preserved. Once these mechanisms are exhausted in the later disease stages, our model predicts that the spike train regularity increases and the fluctuations in the population activity decrease. Such phenomena (weakening of synaptic coupling decreasing the CV) have also been found in other computational studies [80, 108]. However, an experimental investigation on the evolution of activity statistics in the brains of AD animal models is, to our knowledge, yet to be performed.

In addition to the effects on the activity statistics, we demonstrate that the loss of synapses results in a reduction of the network’s sensitivity to small perturbations, which goes hand-in-hand with an increase in linear stability. In the presence of unlimited firing rate homeostasis, the perturbation sensitivity, as well as all other dynamical network characteristics, are preserved, even if the extent of synapse loss is substantial. In addition to the dynamical features, the total synaptic contact area, which is decreased in the AD network due to synapse loss, is largely retained. If the homeostatic synapse growth is limited, the network dynamics as well as the total synaptic area are preserved as long as the firing rate can be maintained. Beyond this point, the network quickly approaches the state of the pathological AD network without homeostasis. The effectiveness of homeostatic compensation investigated in this study provides a possible explanation for why morphological disease-related changes in the brain (e.g. synapse loss) precede any clinically recognizable cognitive deficits by years or even decades [31]. The fact that homeostasis is able to recover all network characteristics is non-trivial because in the homeostatic network with few but strong EE synapses, the statistics of the synaptic input (mean and variance) is altered with respect to the intact reference network with many weak EE synapses.

In order to investigate this observation further, we analyze the linear stability characteristics of the network and find a unique dependency of the network’s spectral radius on the firing rate under unlimited homeostasis. Previous theoretical studies have shown that simple recurrent neuronal networks exhibit optimal computational performance for a variety of tasks if they operate in a regime where small perturbations are neither amplified nor instantly forgotten, i.e. close to the edge of chaos [49–54]. Here, we regard the network’s sensitivity to a small perturbation as an indicator of its computational performance in a broad sense. Assuming that a healthy network acts close to the edge of chaos, our results suggest that the EE synapse loss observed in AD moves the dynamics of the network away from that point towards a less sensitive regime with stable dynamics.

This key prediction of our study can be tested experimentally in animal models by analyzing time series of recorded neuronal activity. The degree of chaoticity can be revealed by the application of metrics such as the power spectrum, autocorrelation function, fractal dimension, Lyapunov exponents (for review, see [108–110]), and the analysis of neuronal avalanches [108, 110, 111]. Our prediction of such experiments would be that the degree of chaoticity only depends on the network’s firing rate regardless of the exact synapse configuration or EI balance (compare Sec. *The interplay of hypo- and hyperactivity and its effect on E/I balance and perturbation sensitivity*).

Whereas our analysis accounts for why the sensitivity to perturbation recovers under unlimited homeostasis, it is notable that the coefficient of variation and the Fano factor of the spike trains are also preserved, suggesting a relationship between the transition from the stable to the chaotic regime and these two network activity characterizations. It has previously been proposed that the transition in spiking neuronal networks from the homogeneous asynchronous state (small sensitivity to perturbation and small CV) to the heterogeneous asynchronous state (high sensitivity to perturbation and high CV) is equivalent to the point where analogous rate networks become chaotic [80, 112]. Such a relationship would also explain our observation that the maintenance of the stability of the linearized network dynamics coincidences with the maintenance of the CV.

Our results raise the question of why a shift towards more stable dynamics would be disadvantageous for the system. From networks that exhibit binary or rate dynamics we know that they are insensitive to perturbation in the input and prone to fading memory (changes in the external input are quickly forgotten, see, e.g., [113, 114]). For spiking networks, such as the one investigated in this study, it has been shown that chaotic dynamics, by allowing the system to be more flexible in responding to new inputs, are beneficial in periodic pattern generation tasks and liquid state computing [45, 115, 116]. In [115], for example, a recurrent network’s internal weights are fixed and initialized to ensure the network operates in a chaotic regime, while synapses feeding back from an external readout are trained with a supervised learning algorithm (FORCE learning). This study demonstrates the benefits of chaotic dynamics for pattern generation tasks. Additionally, in the absence of output feedback (the classical *reservoir computing* paradigm), whereby the recurrent connections are fixed and only the weights of the connections from the recurrent network to the readout units are trained, it has been shown that stable dynamics impairs computational performance in simple classification tasks [45, 117].

On the other hand, insensitivity to small perturbations makes the system less susceptible to disruption by noise and is a prerequisite for the formation of stable attractors, which have been frequently used as a memory storage mechanism in neuronal networks (e.g., [118]). However, more recent recordings in prefrontal and association cortices reveal that single cells exhibit complex and variable dynamics with respect to stimulus representation [119], which neither supports the hypothesis of stable attractors nor points to a network dynamics in the stable regime. Computational studies that have investigated the memory capacity whilst taking heterogeneous neural dynamics into account have found that memory formation succeeds in a chaotic regime [120, 121] or with an embedding of stable subspaces in chaotic dynamics [122]. In addition, the construction of associative memory based on unstable periodic orbits of chaotic attractors has been suggested as a possible way of increasing memory capacity [123]. Thus, stable dynamics appear to be at odds with experiments and might even prove disadvantageous for memory formation. Additionally, concrete links between the stability of network dynamics (particularly the drift towards more sensitive regimes, such as those emerging from gradual synapse loss) and processing capabilities under complex, cognitively-relevant computations are lacking and ought to be established in the future.

On the cognitive level, these results suggest that, as homeostatic compensation mechanisms begin to fail, the shift of dynamics towards the stable regime would cause a decrease in performance within a variety of domains. For example, deficits in memory, known to primarily affect recent experiences of the AD individual, could be accounted for by the hypothesis that chaotic dynamics are needed to form new attractors [121]. In addition, very stable dynamics hinder the transition from one attractor to another, which might explain the difficulties of AD patients to perform task switching and dual task processing [124, 125]. Finally, the observation that AD patients often show repetitive speech and actions [126] might be explained by difficulties in moving away from the corresponding attractor state.

So far, only a few other studies on this abstraction level exist that investigate the relationship of the physical symptoms of Alzheimer’s disease to its cognitive deficits. With respect to memory, the effect of synapse loss and compensation through maintaining the TSCA has been investigated in an associative memory model [39–41]. In accordance with our results, the impairment of memory retrieval due to (excitatory) synapse loss was shown to be successfully compensated by restoring the TSCA, if the restoration occurs sufficiently quickly. The effect of the restoration on the firing rate was not explicitly shown. Although these studies demonstrated that homeostasis via synaptic up-regulation can retain memory performance, they lack a systematic investigation of different network parameters and do not provide an analytical explanation for the results.

The dynamical and computational consequences of intrinsic and synaptic-scaling based homeostatic processes have been investigated in previous studies [32, 127]. Consistent with our findings on the effect of firing-rate homeostasis in the presence of synapse loss, [127] showed that homeostatic intrinsic plasticity helps maintaining a given (chaotic) working point in the presence of external perturbations (constant external inputs). It prevents recurrent neuronal networks from drifting into a regular (non-chaotic) regime and thereby improves input separability. Similarly, [32] demonstrated that homeostatic synaptic scaling can compensate for a partial deafferentation (loss of external inputs) and maintain the macroscopic network dynamics, provided the degree of deafferentation does not exceed a certain critical level. Above this critical level, their model networks develop into a state dominated by slow oscillations, dense activity and bursting, similar to the effects observed in several CNS disorders. A direct comparison with our study is difficult as the networks in [32] are small (100 neurons in total, each excitatory neuron projecting to 5 excitatory and 2 inhibitory cells), the connectivity is distance-dependent, and neurons are described by two-compartment conductance-based models. Moreover, [32] investigated the effect of changes in the external input, whereas we focus on a loss of recurrent connectivity. In our study, we show that homeostatic synaptic scaling preserves firing rates, linear stability, sensitivity to small perturbations, as well as the degree of firing irregularity (CV) and synchrony (Fano factor). We do not observe any low-frequency oscillatory behaviour. It remains a task for future studies to investigate whether the effects observed in [32] generalize to the type of network studied here.

We complement our numerical results by an analytical approach to gain an intuitive understanding of the mechanisms underlying the recovery of the perturbation sensitivity (and hence, computational performance) by firing rate homeostasis. To study the linear stability characteristics of the network, we apply mean-field theory, similar to the approach used by [80]. Note that we do not claim that a loss of linear stability coincides with the transition from stable to chaotic dynamics [54, 81–84], as observed in large autonomous random networks of analog neurons [79]. Rather, we exploit that the linear stability characteristics follow a similar trend as the perturbation sensitivity. Assessing the linear stability characteristics relies on the knowledge of the effective connection strengths, i.e. the number of excess response spikes evoked by an additional input spike in the presence of synaptic background activity. This effective connectivity can be obtained experimentally (see, e.g., [128, 129]), or, for a specific neuron and synapse model, numerically (see, e.g., [130–132]). For simplified models, such as the leaky integrate-and-fire neuron studied here, it can be calculated analytically under simplifying assumptions (diffusion approximation; [133, 134]). However, we note that the preservation of linear stability by firing-rate homeostasis is due to the approximately exponential shape of the gain function. It remains to be investigated whether our results can be generalized to other types of neurons with different gain functions. Our theoretical analysis exposes the working-point dependence of the effective weights as the essential mechanism underlying the recovery of linear stability by firing rate homeostasis: on the one hand, the upscaling of EE synaptic weights required for maintaining the firing rates contributes to a destabilization of the network dynamics. On the other hand, the increase in synaptic weights leads to an increase in the variance of the synaptic-input fluctuations, which, in turn, reduces the neurons’ susceptibility to modulations in the presynaptic input, and therefore stabilizes network dynamics. Note that a similar effect has been described in [135].

Both our theoretical approach and our numerical simulations are predicated on random network connectivity. This randomness, however, neglects basic structures of brain areas that undergo severe pathologic changes in the course of AD progression, e.g. hippocampus, prefrontal cortex and cerebellum. These regions are all organized into layers, and have local connectivity structure comprising special features such as distant-dependence [136] or clustered synaptic connectivity [137]. The precise impact of such structural aspect is not yet clear. From the study of [50], we can conclude that, for networks with distant-dependent connectivity, the systematic increase of synaptic weights makes the network more sensitive, as we also observed in this work. However, a more systematic investigation of different network connectivity structures is needed to determine whether our results are robust with respect to different connectivity constraints.

The results reported in this study are based on a model of AD where synapse loss and synaptic scaling are confined to connections between excitatory neurons (EE). The motivation for restricting our investigation to the loss of EE connections is that this appears to be a prominent feature in many cortical areas [7, 71, 105]. Evidence that other types of synapses are also damaged in the course of the disease has been gathered from several mouse models. For example, inhibitory synapses from neurons in the entorhinal cortex to excitatory CA1 hippocampal neurons have been found to be selectively degenerated in AD mice [95]. Our mean field theoretical results suggest that a global unspecific synapse loss affecting all types of connections (EE, EI, IE, II) leads to noticeable changes in firing rates and linear stability characteristics, but only for higher levels of synapse loss (more than 50%; see, S2 Fig of Supplementary Material). In this scenario, a recovery of firing rates by a synapse unspecific scaling of synaptic weights largely preserves the linear stability characteristics, similar to our findings obtained for a EE synapse loss and EE synapse scaling. This suggests that the commonly reported scaling of EE synapses may well be a mechanism the brain employs to compensate for alterations in dynamical characteristics that are induced by other types of synapse loss.

Although synapse loss correlates best with the cognitive decline observed in AD, by focusing on this aspect, the current study neglects other physical manifestations of AD such as neuron death and alterations of intrinsic neuronal properties [138–142]. These phenomena would affect both inhibition and excitation in the network, so the changes of the resulting firing rate may well be non-monotonic, unlike in our model, having unpredictable effects on the the computational properties. Alternatively, they might be entirely unaffected: in a computational study, [143] showed that under some circumstances, a network can compensate for neuron loss without the need for additional homeostasis mechanisms by adjusting neuronal transfer functions. The contribution of intrinsic neuron properties to the claimed hyperexcitability of inhibitory neurons observed in AD has been previously investigated in a computational study by [144]. Whereas the interplay of such properties with synaptic loss and homeostasis are beyond the scope of the current work, our model could be extended to incorporate these aspects. However, there is as yet no consensus on which cell type shows hyperactivity [145] or hypoactivity [146]; which moreover may vary over the course of the disease [91, 147].

Analogously to our focus on synaptic loss in EE connections, we also restricted our investigation of firing rate homeostasis to EE synapse growth. This is motivated by the findings that intense synaptic upscaling is observed in AD and that an increase of excitatory-excitatory connections has been reported as a main compensation mechanism that increases the firing rate in hippocampal and cortical neurons after an artificially induced decrease in activity (e.g. by blocking sodium channels (TTX) or glutamatergic synapses or AMPAR [148–154]). In Sec. *Effect of local homeostasis on perturbation sensitivity* we show that, regardless of the type of homeostatic regulation (global or local), sensitivity is a unique function of the firing rate. For local homeostasis, we only focus on postsynaptic regulation and neglect presynaptic adaption such as changes in release probability and the size of vesicle pools [34, 152, 154–164]. Since our results are robust with respect to the two types of homeostasis we investigated here, we expect that our results are also robust with respect to the model of synaptic plasticity. Apart from synaptic scaling, other mechanisms that increase the network’s firing rate could also be considered, e.g. changes in current flow of ions (e.g., [165, 166]) or moving the spike-initiation zone [167].

In order to understand the complexity of Alzheimer’s disease, it is important to study the effects of the different observed morphological alterations caused by AD, their corresponding homeostatic responses and, crucially, how they interfere with each other.

The findings of our study suggest that homeostatic synaptic scaling might be an attractive target for drug development. However, some caution is required. Firstly, as discussed above, during early AD the neuronal activity seems to be increased, followed by a decrease. Thus, enhancing EE synaptic scaling at the very beginning of AD manifestation could even accelerate the progression of the disease. In the later stages of the disease, supporting synaptic scaling might be beneficial, stabilizing the cognitive performance. Within this context, there are a variety of molecular substrates that regulate synaptic scaling, and which show altered expression patterns in AD, that could be considered as treatment targets, for example MSK1, PSD-95, BDNF, Arc, Calcineurin, CaMK4 and Cdk5 (for reviews see [168]). A major challenge is to determine whether the altered concentrations of these substrates are a consequence of direct AD pathology, or arise as an attempt of the organism to counteract pathology, or even a mixture of both. Thus, in addition to more comprehensive modelling investigations, further research on the exact timeline of morphological changes and their functional implications is needed to identify promising therapeutic targets.

So far, we have related synapse loss and homeostasis solely to observations made in Alzheimer’s disease. Thus, the present study shows that certain cognitive deficits in Alzheimer’s disease may be attributed to changes in the stability characteristics of neuronal network dynamics. Its central aim is to contribute a deeper insight into the relationship between disease-related alterations at the structural, the dynamical and the cognitive levels. The findings of this study are also applicable in an entirely different context: in the face of limited computational resources, neuronal network models are often downscaled by reducing the number of nodes or the number of connections while increasing their strength. This downscaling has limitations if dynamical features such as the temporal structure of correlations in the neuronal activity are to be maintained [169]. The present work demonstrates that certain functional characteristics such as the sensitivity to perturbations or the classification performance can be largely preserved, if the synaptic weights are not limited by biological constraints. This insight may be particularly relevant for cognitive-computing applications based on recurrent neuronal networks implemented in neuromorphic hardware [170]. Here, the realization of natural-density connectivity and communication constitute a major bottleneck, whereas the strength of connections is hardly limited.

## Methods

### Network model

#### Network description

The network consists of *N* = *N*_E_ + *N*_I_ identical leaky integrate-and-fire neurons, subdivided into a population of *N*_E_ = 1000 excitatory and a population of *N*_I_ = *N*_E_/4 inhibitory neurons. In the intact reference network, each excitatory (inhibitory) neuron receives local excitatory inputs from *K*_EE_ = *ϵN*_E_ (*K*_IE_ = *ϵN*_E_) randomly selected excitatory neurons, and inhibitory inputs from *K*_EI_ = *ϵN*_I_ (*K*_II_ = *ϵN*_I_) randomly selected inhibitory neurons. In addition, the neurons in the local circuit are driven by external excitatory inputs modeled as an ensemble of *p* Poissonian spike trains with constant rate *ν*_X_. Each of these external spike trains is sent to a subset of $K_{\text{X}}^{\text{out}}$ randomly selected (excitatory and inhibitory) neurons in the network. Synaptic interactions are implemented in the form of stereotype exponential postsynaptic currents with a time constant *τ*_s_. The strength *J*_*ij*_ of interaction between two neurons *j* and *i*, the synaptic weight, is parameterized by the amplitude of the postsynaptic potential of neuron *i* evoked by an incoming spike from neuron *j*. In the reference network, all excitatory connections and all inhibitory connections, respectively, have equal synaptic weights, i.e. *J*_EE_ = *J*_IE_ = *J* and *J*_EI_ = *J*_II_ = −*gJ*. The greater number of excitatory inputs is compensated by a larger amplitude of inhibitory synaptic weights (*g* = 6).

Unless stated otherwise, the network simulations are repeated for *M* = 10 random realizations of network connectivity, initial conditions and external inputs for each parameter configuration. A detailed description of the network model components, dynamics and parameters is given in the Supplementary Material (S1–S4 Tables). Simulations were performed using NEST (www.nest-simulator.org) version 2.10.0 [171]. All scripts for generating and plotting the data are online at http://doi.org/10.5281/zenodo.3752777.

#### AD implementation

Unless stated otherwise, AD is implemented by a systematic reduction of excitatory synapses to excitatory neuron (EE synapses), by reconnecting the same network with a smaller EE in-degree *K*_EE_. All other in-degrees (*K*_IE_, *K*_EI_, *K*_II_) are preserved. In Sec. *The interplay of hypo- and hyperactivity and its effect on E/I balance and perturbation sensitivity*, we moreover study the effects of hyperactivity, which we induce by systematically increasing the weight *J*_EE_ of EE synapses such that *J*_EE_ > *J*_IE_ (both in the presence and absence of synapse loss).

#### Global homeostasis

In the presence of global firing-rate homeostasis, the removal of EE connections is compensated by increasing the weights *J*_EE_ of the remaining EE synapses such that the time and population averaged firing rate $\nu ={\left(NT\right)}^{-1}\sum_{i=1}^{N}\int_{0}^{T}dt\;s_{i}\left(t\right)$ is preserved. Here, *s*_*i*_(*t*) denotes the spike train generated by neuron *i* (see below), and *T* = 1s the simulation time. The upscaling of the EE weights *J*_EE_ is performed through bisectioning with an initial weight increment Δ*J*_EE_ = *J*_EE_. The algorithm is stopped once the population averaged firing rate *ν* matches the rate of the corresponding intact reference network up to a precision of 0.5%. In the case of limited homeostasis, *J*_EE_ is set to 1.2⋅*J* if the solution of the bisectioning exceeds 120% of the reference weight *J*. The weights *J*_IE_, *J*_EI_ and *J*_II_ of all other connections are not changed by the firing rate homeostasis.

#### Local homeostasis

The local homeostasis implementation used in Sec. *Effect of local homeostasis on perturbation sensitivity* employs a form of structural plasticity which aims at maintaining the cell-specific time-averaged firing rates in the presence of synapse loss. The details of the structural plasticity mechanism are described in [101]. Associated parameter values are given in the S4 Table in the Supplementary Material. Briefly: The employed structural plasticity model generates new or removes existing incoming excitatory connections locally, i.e. for each excitatory neuron, based on the intracellular calcium concentration [Ca^2+^](*t*). The intracellular calcium concentration [Ca^2+^](*t*) is modeled as the low-pass filtered spiking activity of the postsynaptic cell (with time constant *τ*_lh_ and calcium intake per spike *β*_lh_). Synapses are generated or removed according to the time dependent local synapse count *z*(*t*). The change in *z*(*t*) is governed by a Gaussian growth curve

$$\begin{array}{c} \frac{dz}{dt}=\kappa_{\text{lh}}[2\cdot \text{exp}{\left(-\frac{\left[{\text{Ca}}^{2+}\right]\left(t\right)-\xi_{\text{lh}}}{\zeta_{\text{lh}}}\right)}^{2}-1] \end{array}$$ (1)

with *ξ*_lh_ = (*η*_lh_ + *ϵ*_lh_)/2 and $\zeta_{\text{lh}}=\left(\epsilon_{\text{lh}}-\eta_{\text{lh}}\right)/\left(2\sqrt{\ln2}\right)$. Here, *κ*_lh_ denotes the growth rate, *ϵ*_lh_ the target calcium concentration (proportional to the firing rate of the postsynaptic neuron), and *η*_lh_ the minimum calcium concentration needed to create new synapses. The synaptic connectivity is updated according to the described dynamics in discrete time steps of size Δ*t*_lh_. Newly formed synapses onto excitatory neurons are randomly and independently assigned to presynaptic neurons in the entire excitatory cell population. Multiple connections between two neurons are allowed. Newly established excitatory synapses are assigned a synaptic weight *J*_lh_. In Sec. *Effect of local homeostasis on perturbation sensitivity* we consider two different cases where new synapses are either weak (*J*_lh_ = 0.1mV) or of the same strength as excitatory synapses in the initial intact network (*J*_lh_ = *J*).

### Synaptic contact area and characterization of network activity

#### Relative total synaptic contact area

We calculate the total synaptic contact area (TSCA) of the EE synapses as the product *J*_EE_ *K*_EE_ of the EE weight *J*_EE_ and the EE in-degree *K*_EE_. The

$$\begin{array}{c} \text{relative}\;\text{TSCA}=\frac{K_{\text{EE}}J_{\text{EE}}}{K_{\text{EE}}^{\text{ref}}J_{\text{EE}}^{\text{ref}}} \end{array}$$ (2)

is given by the ratio of the TSCA of the neurodegenerated network (reduced in-degree *K*_EE_) and the TSCA of the corresponding intact reference network (full in-degree *K*_EE_) with identical weights *J*_IE_, *J*_EI_ and *J*_II_.

#### Spiking activity

We represent the spike train *s*_*i*_(*t*) = ∑_*k*_ *δ*(*t* − *t*_*i*,*k*_) of neuron *i* (*i* ∈ [1, *N*]) as the superposition of Dirac-delta functions centered about the spike times *t*_*i*,*k*_ (*k* = 1, 2, …). The spike count *n*_*i*_(*t*; *b*) is given by the number of spikes emitted in the time interval [*t*, *t* + *b*]. For subsequent analyses, we further compute the low-pass filtered spiking activity *x*_*i*_(*t*) = (*s*_*i*_ * *h*)(*t*) of neuron *i* as the linear convolution of its spike train *s*_*i*_(*t*) with an exponential kernel *h*(*t*) = exp(−*t*/*τ*_f_)Θ(*t*) with time constant *τ*_f_ and Heaviside step function Θ(*t*).

#### Average firing rate

The time and population averaged firing rate $\nu ={\left(NT\right)}^{-1}\sum_{i=1}^{N}n_{i}\left(0;T\right)$ is given by the total number $\sum_{i=1}^{N}n_{i}\left(T\right)$ of spikes emitted in the time interval [0, *T*], normalized by the network size *N* and the observation time *T* = 10s.

#### Fano factor

As a global measure of spiking synchrony, we employ the Fano factor

$$\begin{array}{c} \text{FF}\left(b\right)=\frac{{\text{Var}}_{t}\left(n\left(t;b\right)\right)}{{\left\langle n(t;b)\right\rangle }_{t}} \end{array}$$ (3)

of the population spike count $n\left(t;b\right)=\sum_{i=1}^{N}n_{i}\left(t;b\right)$ for a binsize *b* = 10ms. 〈*n*(*t*; *b*)〉_*t*_ and Var_*t*_(*n*(*t*; *b*) denote the mean and the variance of the population spike count *n*(*t*; *b*) across time, respectively. Here, we exploit the fact that the variance of a sum signal *n*(*t*) is dominated by pairwise correlations between the individual components *n*_*i*_(*t*), if the number *N* of components is large (see, e.g., [172, 173]). Normalization by the mean 〈*n*(*t*; *b*)〉_*t*_ ensures that FF(*b*) does not trivially depend on the firing rate or the binsize *b*. For an ensemble of *N* independent realizations of a stationary Poisson process, FF(*b*) = 1, irrespective of *b* and the firing rate. In this work, an increase in FF indicates an increase in synchrony on a time scale *b*.

#### Coefficient of variation

The degree of spiking irregularity of neuron *i* is quantified by the coefficient of variation CV_*i*_ = SD_*k*_(*τ*_*i*,*k*_)/〈*τ*_*i*,*k*_〉_*k*_ of the inter-spike intervals *τ*_*i*,*k*_ = *t*_*i*,*k*_ − *t*_*i*,*k*−1_, i.e. the ratio between the standard deviation SD_*k*_(*τ*_*i*,*k*_) and the mean 〈*τ*_*i*,*k*_〉_*k*_. For a stationary Poisson point process, CV_*i*_ = 1, irrespective of its firing rate. CV’s larger (smaller) than 1 correspond to spike trains that are more (less) regular than a stationary Poisson process. We measure CV_*i*_ over a time interval *T* = 10s, and report the population average $\text{CV}=N^{-1}\sum_{i=1}^{N}{\text{CV}}_{i}$.

#### EI-balance

We define the EI-balance *B* for each neuron as the ratio of the sums of the incoming currents from excitatory (*I*_*E*_) and inhibitory inputs (*I*_*I*_). Thus, the averaged EI– balance for excitatory neurons is *B*_*E*_ = *I*_*EE*_/*I*_*EI*_ with *I*_*EE*_ = *J*_EE_ ⋅ *K*_EE_ ⋅ *ν*_*E*_ and *I*_*EI*_ = *J*_EI_ ⋅ *K*_EI_ ⋅ *ν*_*I*_ and, accordingly, for inhibititory neurons *B*_*I*_ = *I*_*IE*_/*I*_*II*_ with *I*_*IE*_ = *J*_IE_ ⋅ *K*_IE_ ⋅ *ν*_*E*_ and *I*_*II*_ = *J*_II_ ⋅ *K*_II_ ⋅ *ν*_*I*_. We define the total EI-balance as the average across all neurons *B* ≔ (*N*_E_ ⋅ *B*_*E*_ + *N*_I_ ⋅ *B*_*I*_)/*N*

#### Sensitivity to perturbation

We examine the sensitivity of a network to a small perturbation in the input spikes by performing two simulations with identical initial conditions and identical realizations of external inputs. In the second run, we apply a small perturbation by delaying one spike in one external Poisson input at time t* = 400ms by δt* = 0.5ms. As a measure of the network’s perturbation sensitivity, we compute the Pearson correlation coefficient

$$\begin{array}{c} R\left(t\right)=\frac{{\left\langle \delta x_{i}\left(t\right)\delta x_{i}^{*}\left(t\right)\right\rangle }_{i}}{\sqrt{{\left\langle \delta x_{i}{\left(t\right)}^{2}\right\rangle }_{i}{\left\langle \delta x_{i}^{*}{\left(t\right)}^{2}\right\rangle }_{i}}} \end{array}$$ (4)

of the low-pass filtered spike responses *x*_*i*_(*t*) and $x_{i}^{*}\left(t\right)$ in the unperturbed and perturbed simulation, respectively, for each time point *t*. Here, *δx*_*i*_(*t*) = *x*_*i*_(*t*) − 〈*x*_*i*_(*t*)〉_*i*_ denotes the deviation of the low-pass filtered spike response *x*_*i*_(*t*) of neuron *i* from the population average 〈*x*_*i*_(*t*)〉_*i*_. ${\langle \ldots \rangle }_{i}=N^{-1}\sum_{i=1}^{N}\ldots$ represents the population average. We define the time-dependent and the long-term perturbation sensitivity as *S*(*t*) = 1 − |*R*(*t*)| (Fig 6, bottom panels) and *S* = *S*(*t*_obs_ = 10s) (Fig 7), respectively. An observation of *S*(*t*_obs_) = 0 indicates that the effect of the small perturbation has vanished, i.e. that the network has stable dynamics and is insensitive to the perturbation. An observation of *S*(*t*_obs_) = 1, in contrast, corresponds to diverging spike patterns in response to the perturbation and thus chaotic dynamics. In dynamical-systems theory and related applications, state differences are typically expressed in terms of the Euclidean distance $D=\sqrt{\sum_{i=1}^{N}{\left[x_{i}\left(t\right)-x_{i}^{*}\left(t\right)\right]}^{2}}$. Here, we employ the (normalized) correlation coefficient *R* instead to avoid (trivial) firing rate dependencies. Note that *D* and *R* are redundant in the sense that both can be expressed in terms of the moments ${\langle x_{i}\left(t\right)x_{i}^{*}\left(t\right)\rangle }_{i}$, 〈*x*_*i*_(*t*)^2^〉_*i*_, ${\langle x_{i}^{*}{\left(t\right)}^{2}\rangle }_{i}$, 〈*x*_*i*_〉_*i*_ and ${\langle x_{i}^{*}\rangle }_{i}$.

### Linearized network dynamics and stability analysis

In the following, we describe the analytical approach to investigate the effect of synapse loss and firing rate homeostasis on the network’s linear-stability characteristics. To this end, we employ results obtained from the diffusion approximation of the leaky-integrate-and-fire (LIF) neuron with exponential postsynaptic currents under the assumption that the synaptic time constant *τ*_s_ is small compared to the membrane time constant *τ*_m_, and that the network activity is sufficiently asynchronous and irregular (mean-field theory; [133, 134, 174]). All parameters that are not explicitly mentioned here can be found in the S1 Table in the Supplementary Material.

#### Stationary firing rates and fixed points

For each parameter set (synaptic weight *J*, extent of synapse loss, different types of firing rate homeostasis), we first identify the self-consistent stationary states by solving

$$\begin{array}{c} \begin{array}{cc} \nu_{\text{E}} & =G(\mu_{\text{E}}\left(\nu_{\text{E}},\nu_{\text{I}}\right),\sigma_{\text{E}}\left(\nu_{\text{E}},\nu_{\text{I}}\right))\\ \nu_{\text{I}} & =G(\mu_{\text{I}}\left(\nu_{\text{E}},\nu_{\text{I}}\right),\sigma_{\text{I}}\left(\nu_{\text{E}},\nu_{\text{I}}\right)) \end{array} \end{array}$$ (5)

for the population averaged firing rates *ν*_E_ and *ν*_I_ of the excitatory and inhibitory subpopulations. Here,

$$\begin{array}{c} G\left(\mu ,\sigma \right)={\left(\tau_{\text{ref}}+\tau_{\text{m}}\sqrt{\pi }\int_{y_{\text{r}}}^{y_{\theta }}du\;f\left(u\right)\right)}^{-1} \end{array}$$ (6)

represents the stationary firing rate of the LIF neuron in response to a synaptic input current with mean *μ* and variance *σ*^2^ in diffusion approximation, with $f\left(u\right)=e^{u^{2}}[1+\text{erf}(u)]$, $y_{\text{r}}=\left(V_{\text{r}}-\mu \right)/\sigma +\frac{q}{2}\sqrt{\tau_{\text{s}}/\tau_{\text{m}}}$, $y_{\theta }=\left(\theta -\mu \right)/\sigma +\frac{q}{2}\sqrt{\tau_{\text{s}}/\tau_{\text{m}}}$ and $q=\sqrt{2}\left|\zeta \left(1/2\right)\right|$ (with Riemann zeta function *ζ*; [133, 134, 174]). For stationary firing rates *ν*_E_ and *ν*_I_ of the local presynaptic neurons, the mean and the variances of the total synaptic input currents to excitatory and inhibitory neurons are given by

$$\begin{array}{c} \begin{array}{cc} \mu_{\text{E}} & =(K_{\text{EE}}{\hat{J}}_{\text{EE}}\nu_{\text{E}}+K_{\text{EI}}{\hat{J}}_{\text{EI}}\nu_{\text{I}}+K_{\text{X}}{\hat{J}}_{\text{X}}\nu_{\text{X}})\tau_{\text{m}},\\ \mu_{\text{I}} & =(K_{\text{IE}}{\hat{J}}_{\text{IE}}\nu_{\text{E}}+K_{\text{II}}{\hat{J}}_{\text{II}}\nu_{\text{I}}+K_{\text{X}}{\hat{J}}_{\text{X}}\nu_{\text{X}})\tau_{\text{m}},\\ \sigma_{\text{E}}^{2} & =(K_{\text{EE}}{\hat{J}}_{\text{EE}}^{2}\nu_{\text{E}}+K_{\text{EI}}{\hat{J}}_{\text{EI}}^{2}\nu_{\text{I}}+K_{\text{X}}{\hat{J}}_{\text{X}}^{2}\nu_{\text{X}})\tau_{\text{m}},\\ \sigma_{\text{I}}^{2} & =(K_{\text{IE}}{\hat{J}}_{\text{IE}}^{2}\nu_{\text{E}}+K_{\text{II}}{\hat{J}}_{\text{II}}^{2}\nu_{\text{I}}+K_{\text{X}}{\hat{J}}_{\text{X}}^{2}\nu_{\text{X}})\tau_{\text{m}}, \end{array} \end{array}$$ (7)

respectively. The coefficients *K*_*pq*_ (*p*, *q* ∈ {E, I}) denote the number of inputs (in-degree) to neurons in population *p* from population *q*, ${\hat{J}}_{pq}=\tau_{\text{s}}C_{\text{m}}^{-1}{\hat{I}}_{pq}$ the corresponding rescaled PSC amplitude, *K*_X_ the number of external inputs for each neuron in the network, and *ν*_X_ the firing rate of the Poissonian external sources. Note that in our network simulations, each external source is connected to a randomly selected subset of $K_{\text{X}}^{\text{out}}$ neurons. As a result, the number *K*_X_ of external inputs each neuron in the network receives is a binomially distributed random number. For the analytical treatment, we neglect this variability and replace *K*_X_ by the average $K_{\text{X}}=pK_{\text{X}}^{\text{out}}/N$. Eqs 5 and 7 are simultaneously solved numerically using the optimize.root() function (method=‘hybr’) of the scipy package (http://www.scipy.org). To ensure that all solutions are found, the fixed-point search is repeated for 30 pairs of initial rates randomly drawn from a uniform distribution between 0 and 50spikes/s. If multiple coexisting fixed points are found, the one with the highest firing rates is chosen for the subsequent analysis.

#### Synapse loss and firing rate homeostasis

**Fig 12:**
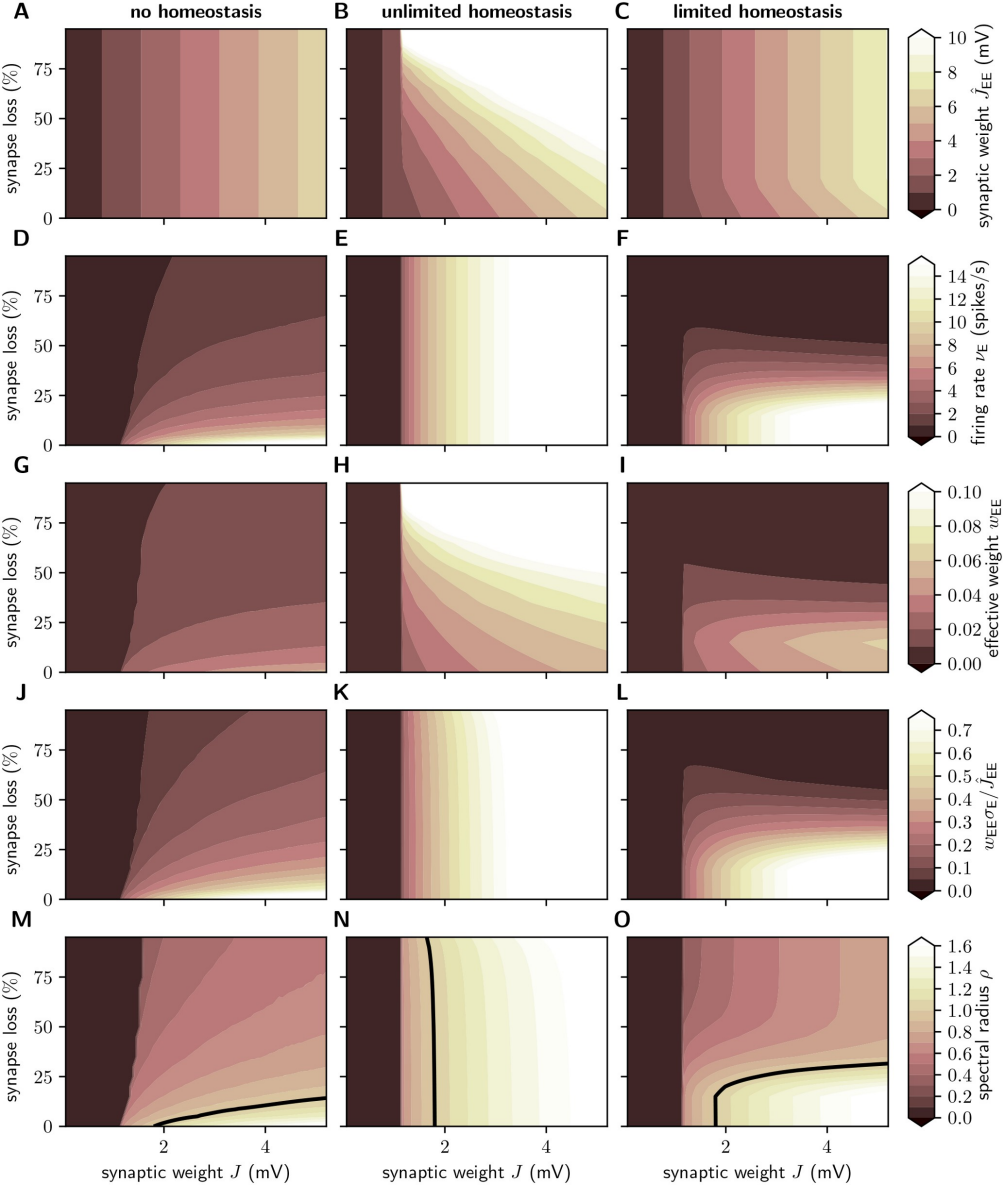
Mean-field theory. Dependence of the synaptic weight ${\hat{J}}_{\text{EE}}$ (**A**–**C**), the average firing rate *ν*_E_ of the excitatory population (**D**–**F**), the effective weight *w*_EE_ of EE connections (**G**–**I**), the ratio $w_{\text{EE}}\sigma_{\text{E}}/{\hat{J}}_{\text{EE}}$ (**J**–**L**), and the spectral radius *ρ* (**M**–**O**) on the synaptic weight *J* and the degree of synapse loss in the absence of homeostatic compensation (left column), as well as with unlimited (middle column) and limited firing rate homeostasis (right column). Superimposed black curves in (M–O) mark instability lines *ρ* = 1. Same parameters as in network simulations (see, S1 and S2 Tables in Supplementary Material).

In this work, Alzheimer’s disease is modeled by removing a fraction of EE synapses, i.e. by reducing the in-degree *K*_EE_. The self-consistent firing rates *ν*_E_ and *ν*_I_ after synapse removal are hence reduced (Fig 12D). In the presence of unlimited firing rate homeostasis, we adjust the weight ${\hat{J}}_{\text{EE}}$ (of the remaining synapses) (Fig 12B) until the excitatory self-consistent firing rate $\nu_{\text{E}}^{\text{ref}}$ of the intact reference network (before synapse removal) is recovered (Fig 12E). To this end, we numerically find the roots of $\nu_{\text{E}}-\nu_{\text{E}}^{\text{ref}}$ by employing again scipy’s optimize.root() function. We repeat the root finding for 30 initial weights randomly drawn from a uniform distribution between ${\hat{J}}_{\text{EE}}^{\text{ref}}$ and $10{\hat{J}}_{\text{EE}}^{\text{ref}}$, where ${\hat{J}}_{\text{EE}}^{\text{ref}}$ denotes the original weight before synapse removal, and keep the solution where $|\nu_{\text{E}}-\nu_{\text{E}}^{\text{ref}}|$ is minimal. For limited homeostasis, the new EE weight is chosen as the minimum of the solution ${\hat{J}}_{\text{EE}}$ and $1.2{\hat{J}}_{\text{EE}}^{\text{ref}}$ (Fig 12C and 12F).

#### Linearized network dynamics and effective connectivity

As shown in [173, 174], networks of spiking neurons can be formally linearized about a stationary state $\nu^{*}=\left(\nu_{1}^{*},\ldots ,\nu_{N}^{*}\right)$ (linear-response theory) and thereby be mapped to an *N*-dimensional system

$$\begin{array}{c} \delta \nu_{i}\left(t\right)=\sum_{j=1}^{N}\left(h_{ij}*\delta \nu_{j}\right)\left(t\right)\;\left(i\in \left[1,N\right]\right) \end{array}$$ (8)

of linear equations describing the dynamics of small firing rate fluctuations $\delta \nu_{i}\left(t\right)=\nu_{i}\left(t\right)-\nu_{i}^{*}$ around this stationary state. The stationary states are determined as the self-consistent solutions of

$$\begin{array}{c} \nu^{*}=\phi \left(\nu^{*}\right), \end{array}$$ (9)

where *ϕ*(***ν***_in_) represents the activation function mapping the vector of stationary input rates ***ν***_in_ to the vector of output rates. The coupling kernel *h*_*ij*_(*t*) represents the firing rate impulse response, i.e. the modulation in the output rate *ν*_*i*_(*t*) in response to a delta-shaped fluctuation in the rate *ν*_*j*_(*t*) of presynaptic neuron *j*.

We refer to the area

$$\begin{array}{c} w_{ij}=\int_{-\infty }^{\infty }dt\;h_{ij}\left(t\right) \end{array}$$ (10)

under the coupling kernel as the effective connection weight. It measures the average number of extra spikes emitted by target neuron *i* in response to a spike fired by the presynaptic neuron *j*, in the context of the background activity determined by the stationary state ν*.

Exploiting the fact that the integral of the impulse response of a linear(ized) system is identical to the long-term limit of its step response, the effective weight

$$\begin{array}{c} w_{ij}={\frac{\partial \phi_{i}\left(\nu \right)}{\partial \nu_{j}}|}_{\nu^{*}} \end{array}$$ (11)

is given by the derivative of the activation function *ϕ*_*i*_ of neuron *i* with respect to the stationary firing rate *ν*_*j*_ of neuron *j*, evaluated at the stationary state ν*. With *ϕ*_*i*_(***ν***) = *G*(*μ*_*i*_(***ν***), *σ*_*i*_(***ν***)) from (6), $\mu_{i}\left(\nu \right)=(\sum_{j=1}^{N}{\hat{J}}_{ij}\nu_{j}+K_{\text{X}}{\hat{J}}_{\text{X}}\nu_{\text{X}})\tau_{\text{m}}$, and $\sigma_{i}^{2}\left(\nu \right)=(\sum_{j=1}^{N}{\hat{J}}_{ij}^{2}\nu_{j}+K_{\text{X}}{\hat{J}}_{\text{X}}^{2}\nu_{\text{X}})\tau_{\text{m}}$, we obtain

$$\begin{array}{c} w_{ij}={\frac{\partial G}{\partial \mu_{i}}\frac{\partial \mu_{i}}{\partial \nu_{j}}|}_{\nu^{*}}+{\frac{\partial G}{\partial \sigma_{i}}\frac{\partial \sigma_{i}}{\partial \nu_{j}}|}_{\nu^{*}}=\frac{{\hat{J}}_{ij}}{\sigma_{i}^{*}}\sqrt{\pi }{\left(\tau_{\text{m}}\nu_{i}^{*}\right)}^{2}(f\left(y_{\theta_{i}}^{*}\right)-f\left(y_{r_{i}}^{*}\right)) \end{array}$$ (12)

as the effective weight of the LIF neuron in the stationary self-consistent state given by ν* [173, 174]. Note that for the result on the right-hand side of (12), we account only for the derivative $\frac{\partial G}{\partial \mu_{i}}$ of *G* with respect to the mean input *μ*_*i*_ (DC susceptibility), but neglect the contribution $\frac{\partial G}{\partial \sigma_{i}}$ resulting from a modulation in the input variance $\sigma_{i}^{2}$. Removal of EE synapses and the resulting decrease in stationary firing rates (Fig 12D) leads to a reduction in the effective weight *w*_EE_ of EE connections (Fig 12G). In the presence of (unlimited) firing rate homeostasis, upscaling of EE synapses (Fig 12B) and the resulting preservation of firing rates (Fig 12E) results in an increase in *w*_EE_ (Fig 12H).

#### Stability analysis

For the LIF neuron with weak exponential synapses [174] as well as for a variety of other neuron and synapse models [130–132], the effective coupling kernel *h*_*ij*_(*t*) introduced in (8) can be well approximated by an exponential function *h*_*ij*_(*t*) = *w*_*ij*_ *τ*^−1^exp(−*t*/*τ*)Θ(*t*) with an effective time constant *τ* and Heaviside function Θ(*t*). With this approximation, (8) can be written in form of an *N*-dimensional system of differential equations

$$\begin{array}{c} \tau \frac{d\delta \nu }{dt}=-\delta \nu +W\delta \nu \left(t\right). \end{array}$$ (13)

Here, ***W*** = {*w*_*ij*_} denotes the *N* × *N* effective connectivity matrix and *δν*(*t*) = (*δν*_1_(*t*), …, *δν*_*N*_(*t*)) the vector of firing rate fluctuations. The system (13) has bounded solutions only if the real parts of all Eigenvalues λ_*k*_ of the effective connectivity matrix ***W*** are smaller than unity, i.e. if Re(λ_*k*_) < 1 (∀*k*). If *ρ* = max_*k*_(Re(λ_*k*_)) > 1, the linearized system is unstable and fluctuations diverge. In the original nonlinear LIF network, an unbounded growth of fluctuations is prevented by the nonlinearities of the single-neuron dynamics. For large random networks where the statistics of the coupling strengths does not depend on the target nodes, the bulk of Eigenvalues {λ_*k*_|*k* ∈ [1, *N*]} of ***W*** is located in the complex plane within a circle centered at the coordinate origin and a radius *ρ* which is determined by the variances of the effective connectivity [175]. A single outlier is given by the Eigenvalue λ_k*_ associated with the Eigenvector ***u***_k*_ = (1, 1, …, 1, 1)^T^, which is given by the mean effective weight. In inhibition dominated networks, the mean synaptic weight and, hence, λ_k*_ are negative. The stability behaviour is therefore solely determined by the spectral radius *ρ*. For a random network composed of *N*_E_ excitatory (j∈E; $N_{\text{E}}=\left|E\right|$) and *N*_I_ inhibitory neurons (j∈I; $N_{\text{I}}=\left|I\right|$) with homogeneous in-degrees *K*_*pq*_ (*p*, *q* ∈ {E, I}) and weights

$$\begin{array}{c} w_{ij}=\{\begin{array}{cc} w_{\text{EE}} & \forall i\in E,\;j\in E,\;\text{connection}\;j\to i\;\text{exists}\;\text{with}\;\text{probability}\;\frac{K_{\text{EE}}N_{\text{E}}}{NN_{\text{E}}}\\ w_{\text{EI}} & \forall i\in E,\;j\in I,\;\text{connection}\;j\to i\;\text{exists}\;\text{with}\;\text{probability}\;\frac{K_{\text{EI}}N_{\text{E}}}{NN_{\text{I}}}\\ w_{\text{IE}} & \forall i\in I,\;j\in E,\;\text{connection}\;j\to i\;\text{exists}\;\text{with}\;\text{probability}\;\frac{K_{\text{IE}}N_{\text{I}}}{NN_{\text{E}}}\\ w_{\text{II}} & \forall i\in I,\;j\in I,\;\text{connection}\;j\to i\;\text{exists}\;\text{with}\;\text{probability}\;\frac{K_{\text{II}}N_{\text{I}}}{NN_{\text{I}}}\\ 0 & \forall i,j,\;\text{connection}\;j\to i\;\text{does}\;\text{not}\;\text{exist} \end{array}, \end{array}$$ (14)

the squared spectral radius is given by

$$\begin{array}{c} \rho^{2}=N_{\text{E}}v_{\text{E}}+N_{\text{I}}v_{\text{I}}=N^{-1}(K_{\text{EE}}N_{\text{E}}w_{\text{EE}}^{2}+K_{\text{IE}}N_{\text{I}}w_{\text{IE}}^{2}+K_{\text{EI}}N_{\text{E}}w_{\text{EI}}^{2}+K_{\text{II}}N_{\text{I}}w_{\text{II}}^{2}). \end{array}$$ (15)

Here, $v_{\text{E}}=w_{\text{EE}}^{2}K_{\text{EE}}N_{\text{E}}/\left(NN_{\text{E}}\right)+w_{\text{IE}}^{2}K_{\text{IE}}N_{\text{I}}/\left(NN_{\text{I}}\right)$ and $v_{\text{I}}=w_{\text{EI}}^{2}K_{\text{EI}}N_{\text{E}}/\left(NN_{\text{I}}\right)+w_{\text{II}}^{2}K_{\text{II}}N_{\text{I}}/\left(NN_{\text{I}}\right)$ denote the variances of the effective connectivity *w*_*ij*_ across the ensemble of target cells (*i* ∈ [1, *N*]) for excitatory (j∈E) and inhibitory sources (j∈I), respectively. Without homeostatic compensation, EE synapse loss leads to a stabilization of the linearized network dynamics, i.e. a decrease in *ρ* (Fig 12M). In the presence of unlimited firing rate homeostasis, the spectral radius *ρ* is preserved (Fig 12N), even if a substantial fraction of EE synapses is removed (Fig 12N). If the homeostatic resources are limited, *ρ* is maintained until the upscaled synaptic weights reach their maximum value (Fig 12O).

#### Preservation of linear stability by firing rate homeostasis

**Fig 13:**
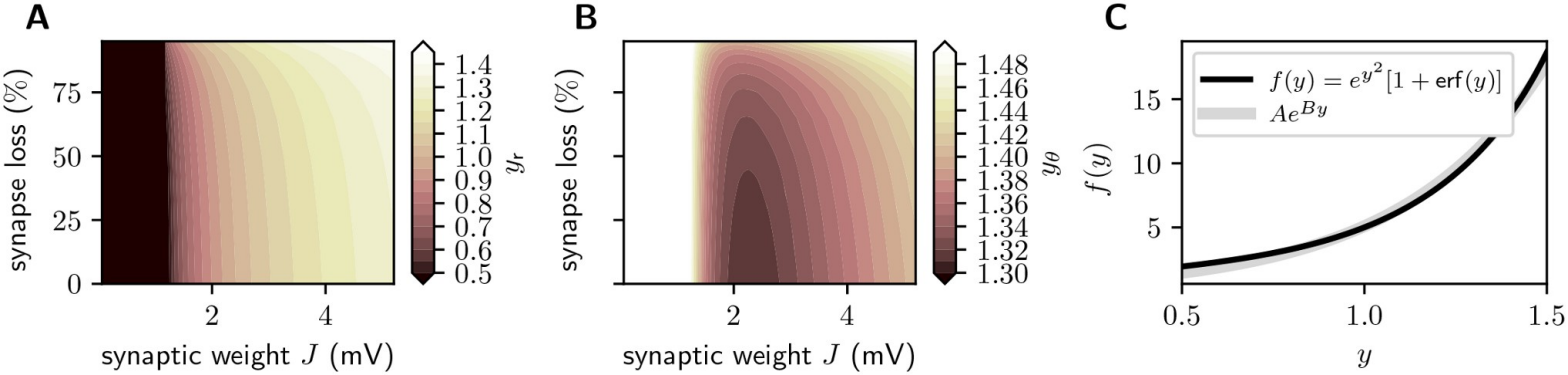
Approximation of $f\left(y\right)=e^{y^{2}}\left[1+\text{erf}\left(y\right)\right]$ by an exponential function. **A**,**B)** Dependence of *y*_r__E_ (A) and *y*_*θ*__E_ (B) on the synaptic reference weight *J* and the degree of synapse loss in the presence of unlimited firing rate homeostasis (mean-field theory). **C)** Graph of $f\left(y\right)=e^{y^{2}}\left[1+\text{erf}\left(y\right)\right]$ (black) and exponential function *Ae*^*BX*^ (gray; *A* = 0.4, *B* = 2.5) fitted to *f*(*y*) in interval *y* ∈ [0.5, 1.5]. Same parameters as in network simulations (see, S1 and S2 Tables in Supplementary Material).

At first glance, it is unclear why firing rate homeostasis preserves the linear stability characteristics as measured by the spectral radius *ρ*. While the stationary firing rates $\nu_{i}^{*}$ are, by definition, kept constant during synapse loss and homeostasis, the input statistics $\mu_{i}^{*}$, $\sigma_{i}^{*}$ (S1D and S1E Fig in Supplementary Material), $y_{r_{i}}^{*}$ and $y_{\theta_{i}}^{*}$ (Fig 13A and 13B) as well as the effective weights *w*_*ij*_ (Fig 12K) are not. To shed light on the mechanisms leading to the preservation of *ρ*, we first note that the factor

$$\begin{array}{c} \sqrt{\pi }{\left(\tau_{\text{m}}\nu_{i}^{*}\right)}^{2}(f\left(y_{\theta_{i}}^{*}\right)-f\left(y_{r_{i}}^{*}\right))≕\eta \left(\nu_{i}^{*}\right) \end{array}$$ (16)

on the right-hand side of (12) is in good approximation uniquely determined by the stationary firing rate $\nu_{i}^{*}$ (Fig 12J–12L). This can be understood by noting that, according to (6), the firing rates are determined by $\int_{y_{{\text{r}}_{i}}^{*}}^{y_{\theta_{i}}^{*}}dy\;f(y),$ and that $f\left(y\right)=e^{y^{2}}\left[1+\text{erf}\left(y\right)\right]$ can be approximated by an exponential function *f*(*y*) ≈ *Ae*^*By*^ for the range of arguments spanned by $y_{r_{i}}^{*}$ and $y_{\theta_{i}}^{*}$ (Fig 13). With this approximation, $\int_{y_{{\text{r}}_{i}}^{*}}^{y_{\theta_{i}}^{*}}dy\;f(y)=B^{-1}[f(y_{\theta_{i}}^{*})-f(y_{{\text{r}}_{i}}^{*})]$. For constant firing rate, $f\left(y_{\theta_{i}}^{*}\right)-f\left(y_{r_{i}}^{*}\right)$ is therefore constant, too, and the effective weight is essentially determined by the ratio ${\hat{J}}_{ij}/\sigma_{i}^{*}$. With $w_{pq}=\eta \left(\nu_{p}^{*}\right){\hat{J}}_{pq}/\sigma_{p}^{*}$ (*p*, *q* ∈ {E, I}), (15) reads

$$\begin{array}{c} \begin{array}{cc} \rho^{2} & =N^{-1}(K_{\text{EE}}N_{\text{E}}\frac{{\hat{J}}_{\text{EE}}^{2}}{\sigma_{\text{E}}^{*2}}\eta^{2}\left(\nu_{\text{E}}^{*}\right)+K_{\text{IE}}N_{\text{I}}\frac{{\hat{J}}_{\text{IE}}^{2}}{\sigma_{\text{I}}^{*2}}\eta^{2}\left(\nu_{\text{I}}^{*}\right)+K_{\text{EI}}N_{\text{E}}\frac{{\hat{J}}_{\text{EI}}^{2}}{\sigma_{\text{E}}^{*2}}\eta^{2}\left(\nu_{\text{E}}^{*}\right)+K_{\text{II}}N_{\text{I}}\frac{{\hat{J}}_{\text{II}}^{2}}{\sigma_{\text{I}}^{*2}}\eta^{2}\left(\nu_{\text{I}}^{*}\right))\\ & =N^{-1}(\eta^{2}\left(\nu_{\text{E}}^{*}\right)N_{\text{E}}\frac{K_{\text{EE}}{\hat{J}}_{\text{EE}}^{2}+K_{\text{EI}}{\hat{J}}_{\text{EI}}^{2}}{\sigma_{\text{E}}^{*2}}+\eta^{2}\left(\nu_{\text{I}}^{*}\right)N_{\text{I}}\frac{K_{\text{IE}}{\hat{J}}_{\text{IE}}^{2}+K_{\text{II}}{\hat{J}}_{\text{II}}^{2}}{\sigma_{\text{I}}^{*2}}). \end{array} \end{array}$$ (17)

According to our network simulations as well as the mean-field theory described above, stationary firing rates of the excitatory and inhibitory subpopulation are identical in the presence of firing rate homeostasis, i.e. $\nu^{*}≔\nu_{\text{E}}^{*}=\nu_{\text{I}}^{*}$. With (7) and assuming that the contribution $K_{\text{X}}{\hat{J}}_{\text{X}}^{2}\nu_{\text{X}}$ of the external drive to the total input variances $\sigma_{\text{E}/\text{I}}^{*2}$ can be neglected (which is the case for the range of parameters considered in this study), we find that the spectral radius

$$\begin{array}{c} \begin{array}{c} \rho^{2}=\frac{\eta^{2}\left(\nu^{*}\right)}{\nu^{*}\tau_{\text{m}}} \end{array} \end{array}$$ (18)

is in good approximation uniquely determined by the stationary firing rate ν* (Fig 8A and S1 Fig in Supplementary Material). A constant firing rate (as achieved by firing rate homeostasis) is therefore accompanied by a constant spectral radius.

### Unspecific synapse loss and homeostasis

In this section, we expand our analysis of the linearized network dynamics towards a network in which all types of synapses (EE,EI,IE,II) are removed. Accordingly, the homeostatic upscaling affects all types of synapses (EE, EI, IE, II) such that the target firing rate is reached by applying the same factor *c* to all synaptic weights and the initial proportion of the different synapse types is kept constant (*c* ⋅ *J*_EE_ = *c* ⋅ *J*_IE_ = *c* ⋅ *J* and *c* ⋅ *J*_EI_ = *c* ⋅ *J*_II_ = −*cg* ⋅ *J* with *c* ≥ 1).

We observe that synapse-unspecific network dilution leads to a drop in firing rate (S2D Fig), but this drop is not as pronounced as if only EE synapses are removed (Fig 12D). For small and moderate degrees of synapse loss, the firing rate changes only little. Upscaling *J* compensates for this and fully restores the firing rates (S2E Fig), even for high levels of synapse loss. In the absence of homeostasis, synapse unspecific network dilution reduces the spectral radius (S2M Fig), but this effect is weaker as if only EE synapses were removed (Fig 12M). For small and moderate degrees of synapse loss, the spectral radius is hardly affected. Upscaling *J* fully recovers the spectral radius in the stable regime (*ρ* < 1). Close to the transition from stable to unstable (*ρ* = 1, black contour line), recovery of the spectral radius is approximately achieved (S2N Fig).

### Different bounds for limited homeostasis

In this section, we investigate the effects of constraining homeostasis to limited degrees of EE synapse growth. If EE synapse growth is limited to only 10%, sensitivity rapidly decreases for synapse losses larger than 10% (S3A Fig). In contrast, if synapses can increase their weights up to 40%, a shift towards the insensitive regime is only observed if more than 30% of the synapses are removed (S3C Fig). Limiting synapse growth to 20% restores the sensitivity as long as a maximum of 20% of the synapses are removed. (=S3B Fig).

## Supporting information

S1 Table Description of the network model according to [176]. (PDF)

S2 Table Network and simulation parameters. (PDF)

S3 Table Parameters for evaluation of spike-train statistics and perturbation sensitivity. (PDF)

S4 Table Parameters of the local homeostasis implementation and of the according sensitivity experiment. (PDF)

S1 FigCanceling of the synaptic-weight variance by the input variance.Dependence of $K_{\text{EE}}{\hat{J}}_{\text{EE}}$ (**A**), $K_{\text{EE}}{\hat{J}}_{\text{EE}}^{2}$ (**B**), $K_{\text{EE}}{\hat{J}}_{\text{EE}}^{2}+K_{\text{EI}}{\hat{J}}_{\text{EI}}^{2}$ (**C**), input mean *μ*_E_ (**D**), input variance $\sigma_{\text{E}}^{2}$ (**E**), and the ratio $\nu_{\text{E}}\tau_{\text{m}}\;\left(K_{\text{EE}}{\hat{J}}_{\text{EE}}^{2}+K_{\text{EI}}{\hat{J}}_{\text{EI}}^{2}\right)/\sigma_{\text{E}}^{*2}$ (**F**) on the synaptic reference weight *J* and the degree of synapse loss in the presence of unlimited firing rate homeostasis (mean-field theory). Note that $\nu_{\text{E}}\tau_{\text{m}}\;\left(K_{\text{EE}}{\hat{J}}_{\text{EE}}^{2}+K_{\text{EI}}{\hat{J}}_{\text{EI}}^{2}\right)/\sigma_{\text{E}}^{*2}$ (F) is very close to unity in all regions where *ν*_E_ > 0 (cf. Fig 12E). Hence, the ratio between the synaptic-weight variance $K_{\text{EE}}{\hat{J}}_{\text{EE}}^{2}+K_{\text{EI}}{\hat{J}}_{\text{EI}}^{2}$ and the synaptic-input variance $\sigma_{\text{E}}^{*2}$ is uniquely determined by the firing rate. Same parameters as in network simulations (see, S1 and S2 Tables in Supplementary Material).(EPS)

S2 FigMean-field theory applied to network with unspecific synapse loss and unspecific synaptic upscaling.We expand our analysis of the linearized network dynamics towards a network in which all types of synapses (EE,EI,IE,II) are removed. Accordingly, the homeostatic upscaling affects all types of synapses (EE, EI, IE, II) such that the target firing rate is reached by applying the same factor *c* to all synaptic weights and the initial proportion of the different synapse types is kept constant (*c* ⋅ *J*_EE_ = *c* ⋅ *J*_IE_ = *c* ⋅ *J* and *c* ⋅ *J*_EI_ = *c* ⋅ *J*_II_ = −*cg* ⋅ *J* with *c* ≥ 1). The figure shows the dependence of the synaptic weight ${\hat{J}}_{\text{EE}}$ (**A**–**C**), the average firing rate *ν*_E_ of the excitatory population (**D**–**F**), the effective weight *w*_EE_ of EE connections (**G**–**I**), the ratio $w_{\text{EE}}\sigma_{\text{E}}/{\hat{J}}_{\text{EE}}$ (**J**–**L**), and the spectral radius *ρ* (**M**–**O**) on the synaptic weight *J* and the degree of synapse loss in the absence of homeostatic compensation (left column), as well as with unlimited (middle column) and limited firing rate homeostasis (right column). Superimposed black curves in (M–O) mark instability lines *ρ* = 1. Same parameters as in network simulations (see, S1 and S2 Tables in Supplementary Material). We observe that synapse-unspecific network dilution leads to a drop in firing rate (**D**), but this drop is not as pronounced as if only EE synapses are removed (Fig 12D). For small and moderate degrees of synapse loss, the firing rate changes only little. Upscaling *J* compensates for this and fully restores the firing rates (**E**), even for high levels of synapse loss. In the absence of homeostasis, synapse unspecific network dilution reduces the spectral radius (**M**), but this effect is weaker as if only EE synapses were removed (Fig 12M). For small and moderate degrees of synapse loss, the spectral radius is hardly affected. Upscaling *J* fully recovers the spectral radius in the stable regime (*ρ* < 1). Close to the transition from stable to unstable (*ρ* = 1, black contour line), recovery of the spectral radius is approximately achieved (**N**).(EPS)

S3 Fig Effect of synapse loss and limited firing rate homeostasis on perturbation sensitivity. Dependence of perturbation sensitivity *S* on the synaptic reference weight *J* and the degree of EE synapse loss for different bounds of limited homeostasis. The different bounds are set such that synaptic weights cannot exceed 110% (**A**), 120% (**B**) and 140% (**C**) of their reference weight. If EE synapse growth is limited to only 10%, sensitivity rapidly decreases for synapse losses larger than 10% (**A**). In contrast, if synapses can increase their weights up to 40%, a shift towards the insensitive regime is only observed if more than 30% of the synapses are removed (**C**). Limiting synapse growth to 20% restores the sensitivity as long as a maximum of 20% of the synapses are removed (**B**).
(EPS)
