# Supplementary material for: Firing rate homeostasis counteracts changes in stability of recurrent neural networks caused by synapse loss in Alzheimer’s disease
Source: PLoS Comput Biol. 2020 Aug 25;16(8):e1007790. doi: 10.1371/journal.pcbi.1007790 (PMC7505475; doi:10.1371/journal.pcbi.1007790)
Supplement: S1 Table — (PDF) [file pcbi.1007790.s001.pdf]

**Summary**	
**Populations**	excitatory population $\mathcal{E}$, inhibitory population $\mathcal{I}$
**Connectivity**	random convergent connections (fixed in-degrees)
**Neuron model**	leaky integrate-and-fire (LIF)
**Synapse model**	exponentially decaying postsynaptic currents, static synaptic weights, fixed delays
**Input**	Poissonian spike trains

**Populations**		
**Name**	**Elements**	**Size**
$\mathcal{E}$	LIF	$N_\mathsf{E} = K/\epsilon$
$\mathcal{I}$	LIF	$N_\mathsf{I} = \gamma N_\mathsf{E} = \gamma K/\epsilon$

**Connectivity**		
**Source**	**Target**	**Pattern**
$\mathcal{E}$	$\mathcal{E}$	random convergent, in-degree K_EE, delay d, weight J_EE
$\mathcal{E}$	$\mathcal{I}$	random convergent, in-degree $K_\mathsf{IE} = K$, delay d, weight $J_\mathsf{IE} = J$
$\mathcal{I}$	$\mathcal{E}$	random convergent, in-degree $K_\mathsf{EI} = \gamma K$, delay d, weight $J_\mathsf{EI} = -gJ$
$\mathcal{I}$	$\mathcal{I}$	random convergent, in-degree $K_\mathsf{II} = \gamma K$, delay d, weight $J_\mathsf{II} = -gJ$
all	all	no self-connections ("autapses"), no multiple connections ("multapses")

**Neuron**	
**Type**	leaky integrate-and-fire (LIF) model
**Description**	dynamics of membrane potential $V_i(t)$ $(i \in \{1, \dots, N\})$<br>• spike emission at t_k^i if $V_i\left(t_k^i\right) \geq \theta$<br>• subthreshold dynamics: $\tau_\mathsf{m}\dot{V}_i = -V_i + R_\mathsf{m} I_i(t) \quad \forall k,\ \forall t \notin \left[t_k^i,\, t_k^i + \tau_\mathsf{ref}\right)$<br>• reset and refractoriness: $V_i(t) = V_\mathsf{r} \quad \forall k,\ \forall t \in \left(t_k^i,\, t_k^i + \tau_\mathsf{ref}\right]$<br>initial membrane-potential distribution at $t = 0$: random uniform between 0 and θ<br>exact integration with continuous spike times in discrete-time simulation [1–3]<br>temporal resolution Δt

**Synapse**	
**Type**	current based synapses with exponential post-synaptic currents (PSCs)
**Description**	$I_i(t) = \sum_{j=1}^{N} \hat{I}_{ij} (\mathsf{PSC} * s_j)(t)$<br>with $\mathsf{PSC}(t) = e^{-t/\tau_\mathsf{s}} \Theta(t)$ and Heaviside function $\Theta(t) = \begin{cases} 1 & t \geq 0 \\ 0 & \text{else} \end{cases}$<br>$\curvearrowright$ post-synaptic potential $\mathsf{PSP}_{ij}(t) = \hat{I}_{ij} \dfrac{R_\mathsf{m}\tau_\mathsf{s}}{\tau_\mathsf{s} - \tau_\mathsf{m}} \left(e^{-t/\tau_\mathsf{s}} - e^{-t/\tau_\mathsf{m}}\right) \Theta(t)$<br>synaptic weight $J_{ij} = \hat{I}_{ij} \dfrac{R_\mathsf{m}\tau_\mathsf{s}}{\tau_\mathsf{s} - \tau_\mathsf{m}} \left(\left[\dfrac{\tau_\mathsf{m}}{\tau_\mathsf{s}}\right]^{\frac{-\tau_\mathsf{m}}{\tau_\mathsf{m} - \tau_\mathsf{s}}} - \left[\dfrac{\tau_\mathsf{m}}{\tau_\mathsf{s}}\right]^{\frac{-\tau_\mathsf{s}}{\tau_\mathsf{m} - \tau_\mathsf{s}}} \right) = \max_t \left(\mathsf{PSP}_{ij}(t)\right)$

**Input**	
**Type**	spike trains modeled as independent realizations of a Poisson point process
**Description**	p independent Poisson spike trains of rate ν_X, each connected to $K_\mathsf{X}^\mathsf{out}$ randomly chosen (excitatory and inhibitory) network neurons

**Realizations**	
**Description**	repetition of network simulations for M random realizations of network connectivity, initial conditions, and external inputs

References

[1] Hanuschkin A, Kunkel S, Helias M, Morrison A, Diesmann M. A general and efficient method for incorporating precise spike times in globally time-driven simulations. PLOS Comput Biol. 2009;5(8):e1000456. doi:10.3389/fninf.2010.00113.

[2] Morrison A, Straube S, Plesser HE, Diesmann M. Exact subthreshold integration with continuous spike times in discrete-time neural network simulations Neural Computation 19 (1), 4749. doi:10.1162/neco.2007.19.1.47.

[3] Rotter S, Diesmann M. Exact digital simulation of time-invariant linear systems with applications to neuronal modeling. Biol. Cybern. 81 (5-6), 381402. doi:10.1007/s004220050570.
