# Supplementary material for: Firing rate homeostasis counteracts changes in stability of recurrent neural networks caused by synapse loss in Alzheimer’s disease
Source: PLoS Comput Biol. 2020 Aug 25;16(8):e1007790. doi: 10.1371/journal.pcbi.1007790 (PMC7505475; doi:10.1371/journal.pcbi.1007790)
Supplement: S2 Table — (PDF) [file pcbi.1007790.s002.pdf]

Connectivity		
**Name**	**Value**	**Description**
K	100	excitatory in-degree (number of excitatory inputs) of reference network
K_{EE}	$5, 10, 20, 30, \ldots, 100$	excitatory-excitatory in-degree
ϵ	0.1	network density
γ	$1/4$	relative size of inhibitory subpopulation
**Neuron**		
**Name**	**Value**	**Description**
τ_{m}	$20\,\mathsf{ms}$	membrane time constant
τ_{ref}	$2\,\mathsf{ms}$	absolute refractory period
C_{m}	$250\,\mathsf{pF}$	membrane capacity
V_{r}	$0.0\,\mathsf{mV}$	reset potential
τ_{s}	$2\,\mathsf{ms}$	time constant of post-synaptic current
θ	$15\,\mathsf{mV}$	spike threshold
**Synapse**		
**Name**	**Value**	**Description**
J	$0.05, 0.1, \ldots, 4.95\,\mathsf{mV}$	EPSP amplitude
g	6	relative IPSP amplitude
d	$1\,\mathsf{ms}$	spike transmission delay
**Input**		
**Name**	**Value**	**Description**
ν_{X}	$750\,\mathsf{spikes/s}$	rate of external Poisson inputs
J_{X}	$0.2\,\mathsf{mV}$	PSP amplitude evoked by external inputs
p	5	number of input sources (spike trains)
$K_{\mathsf{X}}^{\mathsf{out}}$	300	number of neurons each input source is connected to (out-degree)
**Simulation**		
**Name**	**Value**	**Description**
T	0.4, 10 or $2000\,\mathsf{s}$	total simulation time
Δt	$0.1\,\mathsf{ms}$	time resolution
M	10	number of random network realizations per parameter configuration
