# Supplementary material for: Firing rate homeostasis counteracts changes in stability of recurrent neural networks caused by synapse loss in Alzheimer’s disease
Source: PLoS Comput Biol. 2020 Aug 25;16(8):e1007790. doi: 10.1371/journal.pcbi.1007790 (PMC7505475; doi:10.1371/journal.pcbi.1007790)
Supplement: S3 Table — (PDF) [file pcbi.1007790.s003.pdf]

Spike-train statistics		
**Name**	**Value**	**Description**
b	$10\,$ms	binsize for evaluation of Fano factor

Perturbation sensitivity		
**Name**	**Value**	**Description**
t^{*}	$400\,$ms	perturbation time
δt^{*}	$0.5\,$ms	perturbation magnitude (time shift of one input spike)
t_{obs}	$10\,$s	observation time
Δt_{f}	$1\,$ms	time resolution of low-pass filtered spiking activity
τ_{f}	$20\,$ms	time constant of low-pass filter $h(t)$
