# Supplementary material for: Firing rate homeostasis counteracts changes in stability of recurrent neural networks caused by synapse loss in Alzheimer’s disease
Source: PLoS Comput Biol. 2020 Aug 25;16(8):e1007790. doi: 10.1371/journal.pcbi.1007790 (PMC7505475; doi:10.1371/journal.pcbi.1007790)
Supplement: S4 Table — (PDF) [file pcbi.1007790.s004.pdf]

Connectivity		
**Name**	**weak synapses**	**strong synapses**
time constant τ_{lh} of calcium trace	10000.	10000.
calcium intake β_{lh} per spike	0.001	0.001
synapse growth rate κ_{lh}	0.01 elements/ms	0.0001 elements/ms
target calcium concentration ϵ_{lh}	0.05	0.05
minimum calcium concentration η_{lh}	0.0	0.0
EPSP amplitude J_{lh} of newly created synapses	$6 \cdot 10^{-4}$ mV	J
EPSP amplitude J_{IE} of IE synapses	$\{0.5\,\mathsf{mV},\ 1.2\,\mathsf{mV},\ 2.2\,\mathsf{mV}\}$	$\{0.5\,\mathsf{mV},\ 1.2\,\mathsf{mV},\ 2.2\,\mathsf{mV}\}$
connectivity update interval Δt_{lh}	500	$500, 1000$
number of random realizations	3	6
simulation time T	$1200\,\mathsf{s}$	$1200\,\mathsf{s}$
time t^* of perturbation	$1170.4\,\mathsf{s}$	$1170.4\,\mathsf{s}$
